# Treatment of Gastrointestinal Disorders—Plants and Potential Mechanisms of Action of Their Constituents

**DOI:** 10.3390/molecules27092881

**Published:** 2022-04-30

**Authors:** Szilvia Czigle, Silvia Bittner Fialová, Jaroslav Tóth, Pavel Mučaji, Milan Nagy, on behalf of the OEMONOM

**Affiliations:** Department of Pharmacognosy and Botany, Faculty of Pharmacy, Comenius University Bratislava, Odbojárov 10, SK-832 32 Bratislava, Slovakia; silvia.bittner.fialova@uniba.sk (S.B.F.); jaroslav.toth@uniba.sk (J.T.); mucaji@fpharm.uniba.sk (P.M.); nagy@fpharm.uniba.sk (M.N.)

**Keywords:** heartburn, dyspepsia, nausea, constipation, diarrhoea, phytotherapy, HMPC, mechanism of action

## Abstract

The worldwide prevalence of gastrointestinal diseases is about 40%, with standard pharmacotherapy being long-lasting and economically challenging. Of the dozens of diseases listed by the Rome IV Foundation criteria, for five of them (heartburn, dyspepsia, nausea and vomiting disorder, constipation, and diarrhoea), treatment with herbals is an official alternative, legislatively supported by the European Medicines Agency (EMA). However, for most plants, the Directive does not require a description of the mechanisms of action, which should be related to the therapeutic effect of the European plant in question. This review article, therefore, summarizes the basic pharmacological knowledge of synthetic drugs used in selected functional gastrointestinal disorders (FGIDs) and correlates them with the constituents of medicinal plants. Therefore, the information presented here is intended as a starting point to support the claim that both empirical folk medicine and current and decades-old treatments with official herbal remedies have a rational basis in modern pharmacology.

## 1. Introduction

The results of the analysis of several archaeological findings suggest that humanity used plants for therapeutic purposes already about 50,000 years ago, during the upper paleolithic [1]. Empirically acquired knowledge of the effects of “medicinal” plants is gradually inclined in individual regions in comprehensive folk medicine systems, ethnomedicine (Ayurveda, Kampo, Traditional Chinese Medicine, etc.). Current European folk medicine has its roots in the period of Greek and Roman Antiques and follow-up medieval knowledge of the Arab world. Of these, the first works were gradually formed with a particular legislative intention and content, e.g., the formal predecessor of pharmacopoeias, “Ricettario fiorentino” [2], or probably the first pharmacopoeia [3]. The allocation of vegetable drugs to the newly built discipline, pharmacognosis [4], ultimately led to the formulation of European drug legislation in herbal drugs and medicines [5], of which the implementer and professional guarantor are the European Medicines Agency (EMA) and its Committee on Herbal Medicinal Products (HMPC) [6]. However, for the crushing majority of herbal drugs, the directive does not require the explanation of their observed effect with the knowledge of their impact on the processes on the cellular and molecular level.

This review article (see Abbreviations (Supplementary Material, Table S1), too) therefore aims to summarize the current pharmacological knowledge for synthetic drugs used in frequently occurring functional gastrointestinal disorders (FGIDs) and to suggest a possible link to the constituents of registered herbal drugs. A similar approach was already taken in 2002 by Thompson Coon and Ernst [7], of course, using the knowledge of the time. The authors of this review are aware that the data presented are often only at the level of in vitro or in animal models [8] and cannot fully explain the complex action of multicomponent herbal drugs (and their bioactive metabolites) on all possible targets in the human body without detailed in vivo, in vitro, and clinical studies (Supplementary Material, Table S2). Thus, the information presented here is intended to be a starting point to support the claim that empirical folk medicine and the current and decades-old therapy with official herbal preparations based on it have a rational basis in modern pharmacology.

## 2. Functional Gastrointestinal Disorders

The current Rome IV Foundation criteria for the classification of functional gastrointestinal disorders are primarily based on symptoms that occur in clinical practice [9]. For the purposes of this review, only the following of the 32 underlying disorders are relevant: functional heartburn (FH), functional dyspepsia (FDY), nausea and vomiting disorders (NVDs), functional constipation (FC), and functional diarrhoea (FDI). The time factor is a prerequisite for a diagnosis of functional impairment: complaints lasting three months, which need not be consecutive, with an onset at least six months before the diagnosis is made. The worldwide prevalence of FGID is about 40% [10], with standard pharmacotherapy being long-lasting [11] and thus economically challenging [12].

Hand in hand with advances in diagnosis has come a more comprehensive perception of FGIDs. Initially, simpler definitions (the absence of organic disease/a stress-related or psychiatric disorder/a motility disorder/a disorder of GI—gastrointestinal—functioning) [13] have been replaced by a definition that views all concomitant health problems as the result of disharmony on the bidirectional brain–liver–gut–microbiota axis. The current definition of FGID or DBGI is also based on this concept (disorders of brain–gut interactions): “It is a group of disorders classified by GI symptoms related to any combination of the following: motility disturbance, visceral hypersensitivity, altered mucosal and immune function, altered gut microbiota, and altered central nervous system (CNS) processing” [9]. This definition not only reflects the diversity of symptoms observed, but it also indicates the complexity of current therapeutic approaches and the use of synthetic drugs acting through a variety of mechanisms of action.

## 3. Herbal Drugs and Their European Legislation

Licensed synthetic drugs for GI diseases discussed here (e.g., omeprazole, metoclopramide, loperamide, or domperidone) can affect more than one target [14]. This causes both desired therapeutic effects and undesired adverse effects [15]. In real therapeutic practice, adverse effects are often the cause of low compliance [16], which may result in the discontinuation of therapy with the prescribed synthetic drug and switching to herbal medicines. The latter has a large number of licensed (WEU) or registered (TU) products just for gastrointestinal (GIT) diseases.

In the introduction mentioned, HMPC is responsible for compiling and assessing scientific data on herbal substances, preparations, and combinations, to support the harmonization of the European market. There are two categories of herbal drugs on her agenda: “well established use” (WEU) and “traditional use” (TU). While the WEU category relates to human herbal medicines that, simplified, meet the same legislative claims as (semi)synthetic drugs, medicines in the TU category are based on another philosophy [5]: (a) they have indications exclusively appropriate to traditional herbal medicinal products which, by virtue of their composition and purpose, are intended and designed for use without the supervision of a medical practitioner for diagnostic purposes or for the prescription or monitoring of treatment; (b) they are exclusively for administration in accordance with a specified strength and posology; (c) they are an oral, external, and/or inhalation preparation; (d) bibliographical or expert evidence to the effect that the medicinal product in question, or a corresponding product has been in medicinal use throughout a period of at least 30 years preceding the date of the application, including at least 15 years within the community; (e) the data on the traditional use of the medicinal product are sufficient; in particular, the product proves not to be harmful in the specified conditions of use and the pharmacological effects or efficacy of the medicinal product are plausible on the basis of long-standing use and experience.

For both of these categories of herbal medicinal products, the HMPC prepares and regularly updates “assessment reports”, which include (if available): data on medical use, non-clinical data (including primary and secondary pharmacodynamic, safety pharmacology, pharmacodynamic interactions, toxicity, genotoxicity, carcinogenity, reproductive and developmental toxicity), clinical data (including clinical pharmacology, clinical efficacy, clinical studies in special populations), clinical safety and pharmacovigilance (including overview of toxicological/safety data from clinical trials in humans, patient exposure, adverse events, serious adverse events and deaths, laboratory findings, safety in special populations and situations, use in children and adolescents, contraindications, special warnings and precautions for use, drug interactions and other forms of interaction, fertility, pregnancy and lactation, overdose, effects on ability to drive or operate machinery or impairment of mental ability, safety in other special situations), and benefit–risk assessment. The only fundamental difference between the two mentioned categories of herbal medicinal products is that, for the WEU category, the pharmacodynamic properties/effects have to be described; for the TU category, although with considerably more herbal drugs, this is not required by the Directive [5].

Herbal medicines consist of at least one herbal drug or its extract containing tens to hundreds of low molecular weight organic molecules in varying proportions and with varying biological activity. The therapeutic effect of herbal medicines is thus the result of their action on molecular and cellular targets in the human body, but also of their mutual interaction (synergy, additivity or antagonism), and of a variable influence on CYP 450, P-gp or OATP activities, respectively [17].

The classification of herbal medicines according to their effect on the body’s organs (e.g., mouth, skin, GIT) is linked to their primary biological activity (e.g., anti-inflammatory, sedative). Therefore, they can be clearly classified in the ATC system (Anatomical Therapeutic Chemical Classification System) in the case of WEU drugs. In the case of TU drugs, the use of ATC classification may be only a formal but useful aid. Thus, in the case of the herbal medicines discussed in this review, the ATC code A (alimentary) will be formally applicable, and for each subgroup, subsequently A02—drugs for peptic ulcer and gastro-oesophagal reflux disease, A03—drugs for functional gastrointestinal disorders, A04—antiemetics and antinauseants, A06—drugs for constipation, and A07—antidiarrhoeals, intestinal anti-inflammatory/anti-infective agents. Therefore, the herbal drugs listed below and their presumed biological effects can be correlated with synthetic drugs from the above therapeutic areas. However, there are currently no synthetic drugs approved in the EU exclusively for appetite enhancement, orexigenics/appetite stimulants (ATC code: A15). Therefore, the theoretical basis of (therapeutic) appetite control will only be discussed in connection with herbal medicines.

## 4. Herbal Drugs and Functional Heartburn

Proton pump inhibitors (PPIs, H^+^/K^+^-ATPase inhibitors omeprazole, esomeprazole, lansoprazole, dexlansoprazole, pantoprazole, and rabeprazole, respectively) and/or H_2_ antagonists (cimetidine, famoditine, nizatidine, and ranitidine, respectively) are used in the routine therapy of functional heartburn. The only official herbal alternatives are preparations of *Salvia officinalis*. In vitro experiments have confirmed the inhibitory effect of the aqueous-ethanol extract of sage leaves on H^+^/K^+^-ATPase; for a concentration of 0.1 mg/mL, it was 46% and for 0.3 mg/mL, up to 66% inhibition. The control (omeprazole, 0.2 mg/mL) had 49% inhibitory activity. In an in vivo experiment with rats after intraduodenal administration, a 67% decrease in gastric juice acidity was observed for omeprazole (40 mg/kg), while for the extract (600 and 1000 mg/kg), the decreases were 54% and 71%, respectively. According to the authors Mayer et al. [18], carnosol is the probable active constituent. A second study linking carnosol to a gastroprotective effect (and thus to a possible influence on the proton pump), in mouse experiments, showed a decrease in the gastric lesion index from the 45.1 ± 8.3 to 16.4 ± 5.4 for lansoprazole (10 mg/kg), respectively to 17.1 ± 8.2 for carnosol (20 mg/kg). Some (semi)synthetic carnosol derivatives (10 mg/kg) were even more active than lansoprazole [19]. As an alternative to synthetic PPIs, molecules that inhibit H^+^/K^+^-ATPase in vitro (Table 1) and are found in many medicinal plants are also being considered.

Hence, licensed herbal medicines from sage leaves as well as plants with higher polyphenol content (as presented in Table 1) have therapeutic potential for suppressing heartburn on the same pharmacological basis as PPIs.

In conclusion, we can state that the mechanisms of action of these plant metabolites correspond well with the mechanisms of action of synthetic drugs used in the therapy of heartburn.

## 5. Herbal Drugs Used in Functional Dyspepsia

Before we address the issue of functional dyspepsia, it is worthwhile to become familiar with the health problem, which is not included in the Rome IV classification, but in terms of the affected organs of the human body, the imbalance of GI hormones as well as therapeutic options is partly related to functional dyspepsia. This health problem is loss of appetite.

Loss of appetite is an eating disorder symptom typical in the elderly and/or as a result of treatment for certain diseases. The major peripheral components of appetite signaling that communicate with the appetite control center include: (a) energy expenditure signaling associated with resting metabolic rate, and (b) neuroendocrine signaling from the gut, pancreas, and adipose tissue. The physiological signaling that regulates appetite is a complex network linking the central and peripheral nervous systems, which influence each other. The appetite control center in the hypothalamus has a stimulatory and inhibitory circuit that affects other areas of the brain, the peripheral nervous system, and endocrine organs. Resting basal metabolic rate has been proposed as a background driver for hunger. Another important and well known determinant of hunger is the hormone ghrelin (GHR), which is released during fasting as a signaling molecule of the gut–brain axis by enteroendocrine cells of the gastrointestinal tract, especially the stomach [24].

The gut–brain axis is also involved in signaling satiety and fullness. The release of the satiety hormones cholecystokinin (CCK) in the proximal intestine, glucagon-like peptide-1 (GLP-1), and peptide YY (PYY) after food ingestion, both in the distal intestine and the colon, as well as neural inputs from the enteric nervous system, indicate the presence of food in the gut lumen. Leptin release from adipose tissue and insulin from the pancreas acts centrally to suppress hunger, with their levels falling during fasting. In summary, aging- and disease-related changes include decreased and altered GHR sensitivity (which reduces hunger), and increased CCK, GLP-1, and fasting or postprandial insulin, increased in postprandial PYY, and finally increased fasting leptin levels, leading to increased feelings of satiety [25]. 

This diversity of compounds produced in a properly functioning GIT suggests why selectively acting appetite-enhancing synthetic drugs (orexigenics/appetite stimulants, ATC code: A15) are not yet approved in the EU, and this health problem is not a separate part of the above-mentioned Rome IV classification. In contrast, the empirical use of medicinal plant preparations in this indication has had an important place in the history of mankind since ancient times. All the plants used contain low molecular weight constituents, bitters, which have different chemical structures but are related by their ability to activate the bitter taste receptors (TAS2R). These receptors are found virtually throughout the human body, including in the GIT. Thus, in the stomach TAS2R1, -3, -4, -5, -7, -9, -10, -13, -14, -16, -19, -20, -30, -31, -38, -39, -40, -41, -42, -43, -46, and -50 were confirmed [26]. 

It is generally believed that bitter substances trigger GHR production after administration, causing first a short-term increase in food intake, and then a long-term decrease. This also correlates with a slowing of gastric emptying and the modulation of gastrointestinal motility [27]. It should be pointed out that bitter agonists stimulate GHR secretion in vitro but inhibit GHR secretion in vivo in humans. These findings suggest that, in vivo, the secretion of other circulating hormones or neural reflexes may mask the local stimulatory effect of bitter agonists on GHR secretion. Therefore, care should be taken to directly extrapolate findings from in vitro studies to clinical applications in vivo [28].

In addition, since women appear to be more sensitive to bitter substances than men, gender may be a factor that explains some conflicting results in addition to dose and route of administration [29]. Last but not least, many single nucleotide polymorphisms have been discovered in TAS2Rs, which may result in different effects among individuals.

Significantly less is known about the effect of bitters on the second orexigenic hormone, motilin (MOT), than for GHR. Close correlations of fluctuations of hunger ratings with fluctuations in MOT plasma levels, but not with GHR plasma levels, were found [30,31]. A decreased plasma MOT level was measured after the intragastric administration of denatonium benzoate in healthy volunteers [29]. The same research group studied quinine hydrochloride influence at the MOT level, too [32], but no study has shown the expression of hTAS2Rs, human bitter taste receptors, on motilin-producing cells with a similar result as for denatonium. Differences in GHR levels were observed: while denatonium had no effect, quinine reduced GHR levels. The authors noted that the difference in various TAS2Rs activation might be an explanation for the different effects of both tested substances on GHR release.

The action of bitters on orexigenic hormones that are formed after the intake of food into the stomach is also not described in detail. Increased CCK excretion was observed after 500 mg of hop (*Humulus lupulus*) extract given after oral intestinal- and gastric-targeted administration [33]. However, in three studies with quinine hydrochloride applied either in an acid-resistant capsule in healthy males and females [34] or by intraduodenal infusion in healthy, lean men, an increase in CCK was not observed [35,36].

Under the same conditions as when studying CCK [33], oral intestinal- and gastric-targeted administration, the increase in plasma level of PYY has been confirmed, but an intraduodenal infusion of quinine [35] or small intestine administration of 100 mg secoiridoids (gentiopicroside 53.9%, sweroside 25.1%, loganic acid 11.5%, swertiamarin 9.5%) [37] turned out to be ineffective. An explanation can be the same as for discrepancies at GHR: single nucleotide polymorphisms of TAS2R, gender, and different receptors activation due to different chemical structures of studied molecules. 

Part of the mechanisms of perception of bitter substances seems to be due to the activation of vagal nerve fibers [38]. Vagal nerve fibers contain receptors for CCK and PYY and terminate near enteroendocrine cells. Since bitters stimulate the secretion of these peptide hormones, this could subsequently activate vagal nerve fibers [39]. 

In addition to GHR and CCK, Ingram et al. [33] also studied GLP-1 and observed its stimulation, but without a more precise quantification of the effect. The effect of quinine hydrochloride on GLP-1 was studied in two experiments. In the first, quinine was administered via the intragastric administration of quinine, in doses of 275 mg and 600 mg, 30 min before a 350 mL mixed nutrient drink intake [40]. Stimulation of GLP-1 was not observed during the first 30 min, but after drinking the drink, its plasma level increased slightly. The same research group later compared the action of 600 mg quinine, either intragastrically 60 min before, or intraduodenally 30 min before, a nutrient drink [41]. For both routes of application, GLP-1 levels were slightly elevated for both compounds studied. However, when quinine was administered intraduodenally at a low dose (75 mg), GLP-1 levels were not affected [35].

In summary, the action of bitters increases dietary intake within the first 30 min of administration, then gradually decreases [42]. Therefore, the recommendation to take herbal medicines “half an hour before a meal” in the relevant EU herbal drug monographs for the treatment of loss of appetite is justified. 

As shown in Table 2, all 13 herbal drugs with EU monographs contain (among other constituents) structural types typical for bitters.

In a more detailed analysis of the above-mentioned structural types of bitters, specific components can be found in individual plants, which are described in many scientific articles. Bitters are subsequently recorded in the BitterDB database [46], where they are described together with the interacting TAS2R subtype. For example, amarogentin, a secoiridoid from *Gentiana lutea*, is an agonist on human TAS2R1, -4, -39, -43, -46, -47, and -50. Except for TAS2R47, the remaining subtypes are found in the stomach and thus can be activated by this substance. Another bitter, camphor, found in the essential oil of *Achillea millefolium*, interacts with TAS2R4, -10, -14 and -47, the first three subtypes of which are found in the stomach. Absinthin from *Artemisia absinthium* is an agonist of TAS2R10, -14, -46, and -47; all of these have been demonstrated in the stomach. One last example: the bitter disaccharide, gentiobiose (6-*O*-β-d-glucopyranosyl-d-glucose) from *Gentiana lutea* activates only TAS2R16, but this is also present in the stomach. The indicated variability of the activable TAS2Rs in the stomach by individual plant constituents, and hence the drugs prepared from them, may largely explain the relevance and benefits of their polycomponent composition in the treatment of loss of appetite.

Herbal medicines used in mild dyspeptic/gastrointestinal disorders represent the largest indication group according to the number of EU monographs (Table 3). Related to the number of plants (and several related essential oils) is the diversity of constituents that could potentially be co-responsible for the therapeutic efficacy of related herbal medicines.

FDY is characterized by one or more of the following main symptoms: epigastric pain, early satiety, and postprandial sensation of fullness. Accessory symptoms (upper abdominal bloating, nausea, belching) can be present, too. In the daily practice two, often overlapping syndromes occur [48]. Frequently deployed FDY therapy with PPIs has an efficacy of only around 14% [49]. As in the case of functional heartburn, plants with H^+^/K^+^-ATPase inhibiting constituents come into consideration as an alternative to synthetic PPIs (see Section 4). A number of studies suggest the disbalance of CCK, GHR, GLP-1, PYY, gastrin (GAS), somatostatin, and secretin in the pathophysiology of FDY, but the studies are small and findings are heterogeneous [50]. In EU authorized propulsives are D_2_ antagonists (metoclopramide, droperidol). They increase an acetylcholine level following in increase in motility of the stomach and proximal small bowel [14,51]. The pain suppression could be ensured by muscarinic receptors antagonists, e.g., by dicycloverine (=dicyclomine) and glycopyrronium bromide, and by drotaverine, a l-type calcium channel inhibitor [14], all authorized in EU [15]. However, some 5-HT_4_ agonists (e.g., cisapride, mosapride and tegaserod) with prokinetic activity were studied for improving FDY, too [52,53,54]. Up to now, none of these have been implemented in clinical practice in the EU.

### 5.1. Inflammation

The European Society for Neurogastroenterology and Motility Guideline does not consider mucosal alterations in the duodenum, including a duodenal inflammation, as a possible cause of symptom generation [55] although eosinophilic infiltration in the upper small intestine may induce gastroduodenal dysmotility [56]. FDY patients developed severe duodenal inflammation, their proinflammatory cytokines were increased, while the expressions of anti-inflammatory ones were decreased [57]. 

Therefore, it is important to consider medications with anti-inflammatory effects in the treatment of FDY. The variety of plant constituents with anti-inflammatory activity is huge (flavonoids, essential oils components, cinnamic acid derivatives, coumarins, diterpenes, triterpenes, saponins, arylalkanones, Table 3). There are multiple mechanisms providing the desired effect and they are broadly similar for the structural types mentioned. For example, flavonoids inhibit histamine release from mast cells, have a suppressive effect on T-cell proliferation, IL-2 synthesis, and secretion of IgA, IgG, and IgM. They also affect the activity of eosinophils, neutrophils, basophils, macrophages, and monocytes. Several flavonoids inhibit PLA2, COX-1, and LOX, inhibit the production of IL-1β and IL-6, and TNF-α. The anti-inflammatory effect of essential oils is mainly mediated by oxidized derivatives of monoterpenes or some phenylpropanoids through inhibition of the expression of inflammatory mediators (LTB4, PGE_2_, IL-1β, TNF-α, TXB_2_) via NF-κB, as well as by inhibition of COX-1. Arylalkanones inhibit the expression of the same inflammatory mediators by the same mechanism as essential oils. It was confirmed that the anti-inflammatory activity is not produced by iridoids on their own, but only by their modified aglycones. The main mechanism of action is the suppression of TNF-α, IL-1β, IL-2, IL-6, inhibition of PGE_2_, LTB4 and IFN-γ. Verbascoside inhibits ICAM-1 expression, suppresses IL-8, IL-12, IFN-γ, iNOS and NO and inhibits PLA2 and LOX-5 [45].

The observation that synthetic PPIs exhibit anti-inflammatory activity [58,59] can also be applied to plant metabolites with PPI activity based on current knowledge (Table 1). Flavonoids are a typical example [60]. However, the other two plant PPIs mentioned, carnosol and verbascoside, also have anti-inflammatory effects [61,62].

### 5.2. Gastrin

GAS plays a very important role in the process of food digestion. In the stomach, it stimulates parietal cells to secrete gastric acid, which is required for the conversion of pepsinogen to pepsin, promotes gastric contractions, and is also involved in gastric emptying [63].

Information on the GAS/gastric acid link to the effect of bitters is still relatively scarce. The mRNA expression of 25 human TAS2Rs in the human gastric epithelium confirmed transcripts for TAS2R7, TAS2R10, TAS2R14, TAS2R43, and TAS2R46. Simultaneously, it was shown that a bitter perception of caffeine in the mouth generates a signal of aversion, which leads, via vagal withdrawal, to inhibition of gastric acid secretion. However, when caffeine reached the stomach, the mentioned secretion intensified. Experiments definitely demonstrated that the application route of caffeine determines its effects on gastric acid secretion through TAS2R43 [64]. More recently, the same research group confirmed that gallic acid interacts with TAS2R4 in the HGT-1 cell model and regulates gastric acid secretion [65].

It should be noted that bitter substances may also increase GAS release by other mechanisms: through A_2_ receptor antagonism (caffeine—in a cellular model) [66] or PPARα activation, e.g., isohumulone and isocohumulone in transient co-transfection studies [67], isomerized hop (*Humulus lupulus*) extract in the C57BL/6N mouse model [68], biochanin A, naringenin, resveratrol [69] or genistein [70], all in the HEK239 cellular model. In addition to direct activation, an indirect effect of bitters on PPARα can also be considered: after TAS2R activation, TRPM5 is part of the signaling cascade in the G cell, which ensures Na^+^ influx into the cell [71]. Sodium stimulates the production of dopamine, which interacts with D_1_ receptor [72]. Its activation leads to a GAS increase secretion that involves the PPARα pathway [73]. 

Any of the above mechanisms in GAS formation may apply to the following bitters from Table 3: sesquiterpenic lactones, iridoids, secoiridoids, diterpenes, saponins, constituents of essential oils, cyanogenic glycosides, chlorogenic acid, cynarine, gentiobiose, gentianose.

### 5.3. Nicotinic Acetylcholine Receptor

The importance of acetylcholine in FDY is mostly attributed to its ability to increase gastric motility and thereby aid gastric emptying [74]. However, the prokinetic effect of acetylcholine (and other similarly acting molecules) itself non-physiologically accelerates gastric emptying by inducing a fasting type of gastroduodenal motility in the postprandial period and is associated with nausea and vomiting [75]. According to recent views, abnormal physiology in FDY may also be associated with excessive GIT motility [76]. 

Bitters-containing plants may also be used in the therapy of FDY. In fact, bitter substances in the signaling cascade TAS2R—PLCβ2—IP_3_/IP_3_R—TRPM5—VGNC—CALHM1 trigger the release of acetylcholine [77] and may ultimately mimic the action of relevant synthetic drugs. However, it must be kept in the mind that this mechanism will only be relevant in the presence of a meal in the stomach, when the orexigenic hormones GHR and MOT are no longer acting. Therefore, even the multi-herbal drugs containing bitter substances (listed simultaneously in both Table 2 and Table 3) are recommended to be taken only after a meal in the treatment of dyspeptic disorders. 

As shown previously in human α4β2 nAChRs stably expressed in HEK tsA201 cells [78], menthol (present in *Mentha* × *piperita*) is a negative allosteric modulator and not a competitive antagonist, as manifested in the case of α7 nAChR [79]. Subsequently, Amato et al. [80] concluded that, in a mouse model, the relaxant effects of menthol could be due to activation of adrenergic neurons, which in turn reduce acetylcholine release via α-adrenergic receptors [81].

Recently, linalool (present in *Cinnamomum verum*, *Gentiana lutea*, and *Pimpinella anisum*), eugenol (present in *Cinnamomum verum*), and citronellal (present in *Melissa officinalis*) have been confirmed to be inhibitors of rat α3β4 nAChRs; although the latter two only weakly [82]. In the case of protopine (from *Fumaria officinalis*), its spasmolytic effect in an isolated guinea pig ileum model was attributed to competitive antagonism to AChR [83]. In the same model, a spasmolytic effect has also been confirmed for e.g., cynaropicrin [84] from *Cynara cardunculus*/*Cynara scolymus*; ursolic acid [85] from *Melissa officinalis*, *Mentha* × *piperita*, *Origanum dictamnus*, *Rosmarinus officinalis* and *Salvia officinalis*; quercetin present in almost all the plants [86] of Table 3; chlorogenic acid [87] from *Achillea millefolium*, *Artemisia absinthium*, *Cynara cardunculus*, *Cynara scolymus*, *Fumaria officinalis*, *Melissa officinalis*, and *Taraxacum officinale*, and finally verbascoside (=acteoside) [88] from *Aloysia citriodora* and *Marrubium vulgare*.

### 5.4. Calcium Channels

L-type calcium channel inhibitors could be used to suppress abdominal pain related to spasms. Flavonoids, present in almost all higher plants, act by both of these mechanisms in different in vitro and in vivo models [89]. In a small rat intestine model, L-type calcium channels were inhibited by the action of marrubenol, present in *Marrubium vulgare* [90]. Spasmolytic action of menthol, a typical constituent of *Mentha* × *piperita*, is associated with the calcium influx blockade through L-type calcium channels as described in samples of human distal colon [91]. For isoliquiritigenin from *Glycyrrhiza* genus plants, a dose-dependent dual effect on GIT transit was confirmed in vivo (mouse model): low doses (0.03 mg/kg and below) were spasmolytic, doses of 3 and 30 mg/kg were spasmogenic, then transit through the intestine was accelerated. However, only low concentrations of isoliquiritigenin acting as a calcium channel blocker are relevant for European therapeutic practice [92]. The spasmolytic effect of gentiopicroside and swertiamarin in isolated guinea pig ileum model, important secoiridoids from *Gentiana lutea*, can be also explained by interaction with calcium influx [93,94].

### 5.5. Serotonin Receptors

5-HT_3_ receptors have been shown to play a role in the regulation of GI motility, secretion processes and pain perception via the gut–brain axis [95].

Over the past two decades, several metabolites of the Table 3 plants have been characterized as negative allosteric modulators: menthol [96] from *Mentha* × *piperita*; citronellol [97] from *Melissa officinalis*; eugenol [97] from *Cinnamomum verum*; 1,8-cineole [97] from *Achillea millefolium*, *Aloysia citriodora*, *Cinnamomum verum*, *Mentha* × *piperita*, *Peumus boldus*, *Rosmarinus officinalis* and *Salvia officinalis*; linalool [98] from *Cinnamomum verum*, *Gentiana lutea*, *Pimpinella anisum*, and boldine [99] from *Peumus boldus*. *Glycyrrhiza glabra*, *Glycyrrhiza inflata* and *Glycyrrhiza uralensis* contain non-competitive 5-HT_3A_ receptors antagonist (liquiritigenin), competitive 5-HT_3A_ ones antagonists (glabridin, licochalcone A) as confirmed by voltage-clamp technique [100]. 6-Gingerol, 8-gingerol, 10-gingerol, 6-shogaol, zingerone and galanolactone from *Zingiber officinale* also inhibit 5-HT_3_ receptors, which has been repeatedly confirmed in vitro [101], in vivo [102,103,104], and even in patients [105,106,107].

Negative modulation or blockade of 5-HT_3_ receptors by these herbal substances may suppress symptoms of dyspepsia similar to what has been described for, e.g., alosetron, an antagonist of these receptors [108].

It has been previously confirmed that GHR (or its active form, acyl ghrelin, AGHR) is involved in proper gastric motility, and thus may be important in the treatment of dyspepsia [109]. A direct increase in AGHR levels in rats was observed after treatment with hesperidin (in *Rosmarinus officinalis*) and isoliquiritigenin (in *Glycyrrhiza glabra*, *Glycyrrhiza inflata* and *Glycyrrhiza uralensis*) via antagonism on 5-HT_2C_ receptors [110]. The prokinetic effect of 10-gingerol (from *Zingiber officinale*) in the rat model is explained by the inhibition of carboxylesterase, causing the degradation of AGHR, whereas in humans, it could be associated with the inhibition of butyrylcholinesterase, e.g., by the action of glycycoumarin (from *Glycyrrhiza glabra*, *Glycyrrhiza inflata*, and *Glycyrrhiza uralensis*) [111].

Non-selective agonism at 5-HT_4_ receptors is implicated in the prokinetic action of metoclopramide. More recently, hesperidin has been found to act at the same receptors in murine small intestinal interstitial cells of Cajal, which spontaneously generate active pacemaker potential, causing electrical and mechanical activity of smooth muscles [112]. This molecule may be another active substance in *Gentiana lutea* and *Rosmarinus officinalis* in FDY therapy, acting by a different mechanism than bitters. 5-HT_4_ receptors are the target of action (among other receptors) in fixed herbal combination product STW 5 (Iberogast^®^) and its two components, ethanolic extracts of *Chelidonium majus* and *Matricaria recutita*, respectively [113].

The link between the central nervous system (CNS) and the enteric nervous system (ENS) is clearly established [114]. The so-called gut–brain axis provides the brain with nutritional, immunological and, to some extent, environmental information, while helping the brain to adapt digestion and locomotion to the current situation outside the body [115]. These assumptions underline the NICE guidelines recommending tricyclic antidepressants (amitriptyline, imipramine) as first-line treatment for patients with FDY in whom constipation is a non-dominant symptom. There is also evidence that SSRIs (escitalopram, fluoxetine, paroxetine) may also be effective in treating gastrointestinal symptoms [116]. Therefore, the use of preparations from *Hypericum perforatum*, which, in addition to inhibiting monoamine oxidase A, also inhibit synaptosomal re-uptake of neurotransmitters, should not come as a surprise [117].

### 5.6. TRP Channels

These channels, which are found throughout the GIT, act as sensors and interfere with a number of physiological functions such as nociception, chemesthesia, mechanosensation, motility, and secretion [118,119]. Therefore, modulation of TRP functionality is also potentially useful in the therapy of GIT diseases. Although the first representative of the 27-member human TRP channel superfamily—TRPC1—was described as early as 1995 [120,121], in real clinical practice (not related to GIT disorders), only the selective TRPV1 agonist, capsaicin, is currently available. The reason for failures in clinical trials for most candidates is precisely their non-selectivity and the different concurrent physiological roles of TRP channels, resulting in frequent significant adverse effects [119] as well as frequent differences in qualitative response between human and animal experiments. The situation is further complicated by the different responses of a given channel to different doses of agonist. Again, capsaicin and TRPV1 are prime examples: a low initial topical dose causes a reduction in nerve responses to subsequent administration of capsaicin, but not to other agonists, the effect is reversible and persists for several hours (=specific desensitization). Conversely, at an initial high dose, subsequent neural responses are reduced not only to capsaicin but also to other agonists (=non-specific desensitisation) [122]. Thus, similar complications can be expected with the natural compounds from the plants in Table 3, but we consider it appropriate to mention their therapeutic potential for FDY.

#### 5.6.1. TRPV1

This channel is probably the most studied of the entire superfamily of human TRPs. In the human GIT, TRPV1 has been confirmed in muscle, arterioles, mucosa, and enteric nerve plexuses. Upregulated TRPV1 has been confirmed in various GIT diseases, including FDY. It detects spice-sensing compounds [123], is part of the pain perception cascade [124,125], affects motility [126,127] and increases blood circulation in the mucous membranes of the oesophagus, stomach, and small intestine [128,129], which is associated with increased mucus production and neutralizing bicarbonate [130,131]. TRPV1 is involved in visceral hypersensitivity in gastrointestinal disorders including dyspepsia, too [132].

It is therefore not surprising that the most information on the influence of plant extracts or their individual constituents on this channel is for pain relief. A sample biphasic pathway for capsaicin was mentioned in the previous paragraph [122]. In the plants of Table 3, another biphasic agonists come into consideration: *Zingiber officinale* and its gingerols, zingerone, shogaols and 6-paradol [133,134]. A difference in in vitro and in vivo activity was observed for zingerone: it induced TRPV1 opening in cultured TG neurons [135], but in an ICR mouse model, this channel was not involved in zingerone-induced inhibition of pacemaker potentiation [136]. Biphasic effect of agonists are essential oils components, too: camphor from *Achillea millefolium*, *Rosmarinus officinalis* and *Salvia officinalis*; carvone from *Carum carvi* and *Mentha* × *piperita*; citral from *Zingiber officinale*; citronellal from *Melissa officinalis*; 1,8-cineole from *Achillea millefolium*, *Aloysia citriodora*, *Cinnamomum verum*, *Mentha* × *piperita*, *Peumus boldus*, *Rosmarinus officinalis* and *Salvia officinalis*; eugenol from *Cinnamomum verum*; geranial from *Aloysia citriodora* and *Melissa officinalis*; myrcene from *Carum carvi*, *Chamaemelum nobile*, *Foeniculum vulgare*, *Juniperus communis* and *Rosmarinus officinalis*; as well as 4,5-dicaffeoylquinic acid from *Achillea millefolium*. Antagonists also have potential: flavonoids (eriodictyol from *Origanum dictamnus*; naringenin from *Althaea officinalis*, *Helichrysum arenarium* and *Origanum dictamnus*; vitexin from *Aloysia citriodora* and the almost ubiquitous quercetin); ursolic acid from *Melissa officinalis*, *Mentha* × *piperita*, *Origanum dictamnus*, *Rosmarinus officinalis*, and *Salvia officinalis* [45,137].

Abbas in his review for ursolic acid states “… had antinociceptive effect in the intracolonic administration of 0.3% capsaicin. Ca^2+^ imaging technique: inhibited Ca^2+^-flux induced by capsaicin in mammalian cells expressing hTRPV1…” [137] and refers to the original paper by Zhang et al. [138]. However, in the experiment described there, a concentration of 100 μM was used. Given the great similarity of the structures of oleanolic acid and ursolic acid (different position of one methyl group, either at C-19 or at C-20), it is very likely that ursolic acid also has dual properties towards TRPV1 similar to oleanolic acid, the latter activating the channel at 30 μM concentration, inhibiting it at 90 μM [139]. Oleanolic acid was found in the following plants of Table 3: *Centaurium erythraea*, *Centaurium majus*, *Centaurium suffruticosum*, *Melissa officinalis*, *Mentha* × *piperita*, *Origanum dictamnus*, *Rosmarinus officinalis* and *Salvia officinalis* [45].

An inhibitory effect on TRPV1 has also been described in a rodent model for liquiritin [140] present in *Glycyrrhiza glabra*, in HET-1A cells for menthol (found in *Mentha* × *piperita*) [141], in animal models (in vivo in rats and ex vivo in mice) for curcumin [142] from *Curcuma longa* and *Curcuma zanthorrhiza*. Capsaicin via TRPV1 triggers increased mucus production (see also Section 5.11) and thus has a gastroprotective effect not only in a cellular model [143], and in rats [144] and in healthy humans [145]. Thus, it is possible that other plant TRPV1 agonists will have a similar effect.

A link between TRPV1—mucus formation—short-chain fatty acids (SCFAs), e.g., acetate, propionate, and butyrate is being considered. A blockade of the channel leads to a decrease in SCFAs and consequently a decrease in mucin formation [146,147]. In the case of reduced TRPV1 functionality, it is possible to increase SCFA levels by the ingestion of indigestible polysaccharides, including mucilages, which are partially degraded by the intestinal microflora into SCFAs, of which acetate and butyrate are thought to be components that activate mucin secretion [148,149]. The sources of suitable polysaccharides are *Althaea officinalis*, *Linum usitatissimum*, *Malva neglecta*, *Malva sylvestris*, and *Matricaria chamomilla* (Table 3). The above mechanism may effectively complement the historically traditional effect of mucilage in dyspeptic disorders—covering the damaged gastric mucosa with a protective layer. The exploitable adhesiveness of plant mucilages to mucus and mucin, respectively, is attributed only to acidic polysaccharides carrying a negative charge on their galacturonan or glucuronan moieties [150,151]. The above-mentioned plants do indeed contain acidic mucilage [45].

#### 5.6.2. TRPA1

This channel has been confirmed in humans in colonic epithelial cells [152], enterochromaffin cells [153], mucosa [154], intestinal myenteric and motor neurons [155], and in human enteric glial cells cultures [156]. 

There is a correlation between TRPA1 and TRPV1 discussed above. It has been confirmed that TRPA1 can act as an enhancer of TRPV1 [157] and vice versa [158] also thanks to the formation of heterotetramers between the two channels [159]. Therefore, the action of TRPA1 agonists or antagonists may also be reflected in the resulting physiological effects attributed to TRPV1 (see Section 5.6.1). However, the results, especially human ones, are considerably fewer than for TRPV1.

Activation of TRPA1 by agonists (in low doses) induces pain sensation, acts pro-inflammatory, triggers vasodilation in the periphery. In relation to FDY, this channel is involved in recognition of various environmental influences; agonists trigger mucosal HCO_3_^−^ and Cl^−^ secretion, affect gut motility a gastric accommodation, mediate the stimulation of 5-HT release from mucosal enterochromaffin cells, facilitate 5-HT and CCK from enteroendocrine cells.

Formally, TRPA1 agonists can be divided into electrophilic and non-electrophilic [160]. Examples of plant electrophilic TRPA1 activators include isothiocyanates (from horseradish and mustard plants), allicin, diallyl disulfide (in garlic) or cinnamaldehyde (*Cinnamomum verum*, see Table 3) [161,162] and curcumin [163] from *Curcuma longa* and *Curcuma zanthorrhiza*. Representatives of non-electrophilic TRPA1 activators contained in the plants of Table 3 include menthol from *Mentha* × *piperita* [164], carvacrol [165] from *Origanum dictamnus*, β-myrcene [165] from *Carum carvi*, *Chamaemelum nobile*, *Juniperus communis* and *Rosmarinus officinalis*, 1,4-cineole [166] from *Cinnamomum verum*, (−)-α-bisabolol [167] from *Matricaria chamomilla*, eugenol [168] from *Cinnamomum verum*, gingerol, 6-shogaol, 6-zingerone [169], 6-paradol [170], all from *Zingiber officinale*, isoliquiritigenin [171] from *Glycyrrhiza glabra*, *Glycyrrhiza inflata* and *Glycyrrhiza uralensis*; geranyl acetate [172] from *Melissa officinalis*, anisaldehyde [173] and anethole [174] from *Foeniculum vulgare* and *Pimpinella anisum*, citral [175] from *Zingiber officinale*, camphor [176] from *Achillea millefolium*, *Rosmarinus officinalis* and *Salvia officinalis*, linalool [177] from *Cinnamomum verum*, *Gentiana lutea* and *Pimpinella anisum*, carnosol and carnosic acid [178] from *Salvia officinalis*.

Many of these compounds act bimodally: at low concentrations they activate TRPA1, at higher concentrations they inactivate it. TRPA1 antagonists ultimately have an effect as agonists at higher doses. In the plants of Table 3 are found: 1,8-cineole from *Achillea millefolium*, *Aloysia citriodora*, *Cinnamomum verum*, *Mentha* × *piperita*, *Peumus boldus*, *Rosmarinus officinalis* and *Salvia officinalis*, borneol [179] from *Achillea millefolium*, *Rosmarinus officinalis* and *Zingiber officinale*, liquiritin [140] from *Glycyrrhiza glabra*, *Glycyrrhiza inflata*, and *Glycyrrhiza uralensis*.

#### 5.6.3. TRPM8

This non-selective cation channel is also known as a thermoreceptor, which is activated at temperatures 8–28 °C [180]. Previously, knowledge on human colonic TRPM8 expression appears was related to pathological conditions, only [181,182]. Recently, it was demonstrated that TRPM8 receptors are also expressed in human distal colon in healthy conditions and that TRPM8 activation by menthol reduces the spontaneous colonic motility [183].

In a sole human subject study administered peppermint oil (containing menthol, a TRPM8 agonist), the authors reduced gastric motor function [184]. The aforementioned menthol is found in *Mentha* × *piperita* (Table 3). Other agonists may have similar effects: 1,8-cineole from *Achillea millefolium*, *Aloysia citriodora*, *Cinnamomum verum*, *Mentha* × *piperita*, *Peumus boldus*, *Rosmarinus officinalis*, and *Salvia officinalis*; 1,4-cineole from *Cinnamomum verum*; menthone [166] from *Mentha* × *piperita*; eugenol [161] from *Cinnamomum verum*; isopulegole [185] from *Mentha* × *piperita*; geraniol [185] from *Zingiber officinale*; camphor [186] from *Achillea millefolium*, *Rosmarinus officinalis* and *Salvia officinalis*; linalool [185] from *Cinnamomum verum*, *Gentiana lutea* and *Pimpinella anisum*; borneol [187] from *Achillea millefolium*, *Rosmarinus officinalis* and *Zingiber officinale*.

In GIT diseases, overexpressed TPRM8 could be inhibited by some plant metabolites [171]: hispidulin from *Salvia officinalis*; glycycoumarin and licochalcone A from *Glycyrrhiza glabra*, *Glycyrrhiza inflata*, and *Glycyrrhiza uralensis* [45].

Curcumin (from *Curcuma longa* and *Curcuma zanthorrhiza*) showed a different behavior: while in human TRPM8 expressed in HEK293 cells, it was inactive [188], in the rat duct and identical cells manifested as an inhibitor [163].

For the other TRP channels in the human GIT, knowledge on the action of metabolites from the plants of Table 3 is scarce. Therefore, only some of the TRP channels are discussed in this article. Moreover, data from animal or cell experiments can thus only serve as baseline information for formulating basic hypotheses about the potential therapeutic effect of the molecules described below.

#### 5.6.4. TRPM7

These channels were found in interstitial cells of Cajal (ICCs) and it was confirmed they are involved not only in the physiology of normal GIT motility, but also in many bowel disorders [189].

However, only two studies are known for the plant contents of Table 3 at this time. For carvacrol, as TRPM7 inhibitor expressed in both HEK cells and CA3–CA1 hippocampal primary cultured cells [190]. Carvacrol occurs in *Origanum dictamnus* (Table 3). 

Quercetin, a known flavonoid, could participate in the treatment of symptoms related to GI motility disorders such as spasm, pain or transit disturbances by the regulation of pacemaker activity studied in cultured murine ICCs via TRPM7 inhibition [191]. Quercetin can be found in almost all plants from Table 3.

#### 5.6.5. TRPC5

These ionotropic channels are predominantly expressed in the brain, but have also been confirmed in rat small intestinal crypts and Caco-2 cells (derived from human colon epithelium), where they are involved in store-operated calcium entry processes [192]. Recently, it has been shown in a rodent model that inhibition of TRPC5 can effectively suppress pain hypersensitivity [193].

TRPC5 inhibitors are also found in some plants of Table 3: kaempferol [194] from *Althaea officinalis*, *Melissa officinalis* and *Origanum dictamnus*, quercetin present almost everywhere [194], isoliquiritigenin [195] from *Glycyrrhiza glabra*, *Glycyrrhiza inflata* and *Glycyrrhiza uralensis*, 6-gingerol, 6-shogaol, and zingerone [169] from *Zingiber officinale*.

### 5.7. Potassium Channels

K2P2.1 channels (KCNK2, TREK-1) are expressed at high levels in excitable tissues, such as the nervous system [196], and smooth muscle [197]. They have been shown to participate in many important physiological processes, including pain perception. Interestingly, they are co-localized with TRPV1 in small DRG neurons of mice [198].

Different monoterpenes, constituents of some essential oils, were studied in an exogenous expression system to measure their impact on human K2P2.1 channel activity. The activation by carvacrol and menthol at the dose 0.3 mM was dose-dependent and statistically significant. Other monoterpenes (thymol, *p*-cumenol, β-citronellol, *p*-cymene, geraniol, cinnamaldehyde, and eugenol) were found to activate this channel, too, but to a lesser extent [199] Thus, K2P2.1 channel activation could be involved in pain perception decrease by using the following plants (or their essential oils) mentioned in Table 3: *Gentiana lutea* and *Origanum dictamnus* (for carvacrol); *Mentha* × *piperita* (for menthol); *Melissa officinalis* (for β-citronellol); *Chamaemelum nobile*, *Origanum dictamnus*, *Peumus boldus* and *Rosmarinus officinalis* (for *p*-cymene); *Zingiber officinale* (for geraniol); *Cinnamomum verum* (for cinnamaldehyde and eugenol) [45]. Arazi’s research group subsequently confirmed a similar activating effect on K2P4.1 (KCNK4, TRAAK), K2P10.1 (KCNK10, TREK-2) and K2P18.1 (KCNK18, TWIK), which are also involved in pain perception [200].

Glabridin, an isoflavan from *Glycyrrhiza glabra*, *Glycyrrhiza inflata* and *Glycyrrhiza uralensis* (Table 3) could be partially involved in analgesic action of mentioned plants by activating the large conductance Ca^2+^-activated K^+^ channels (KCa1.1 = KCNMA1 = BK) in rat and mice pain models [201]. The same effect was observed with curcumin in the HEK293 cells overexpressing exogenous BK channels and A7r5 cells with endogenous BK, respectively [202].

Curcumin (from *Curcuma longa* and *Curcuma zanthorrhiza*) may also contribute to the protection of the gastric mucosa by activating the NO—cGMP—KATP cascade as described in vivo with rats [203]. In contrast, quercetin-induced relaxation of human gastric smooth muscle occurs directly via K^+^-ATP channels and independent of the NO pathway [204].

### 5.8. Sodium Channels

Pain in the GIT can also be suppressed by the action of *Glycyrrhiza glabra*, *Glycyrrhiza inflata* and *Glycyrrhiza uralensis* (Table 3), or its component isoliquiritigenin, which inhibits Nav1.7 [205] and Nav1.8 in CHO cells [206]. Inhibition of Nav1.8 also shows 6-gingerol and 6-shogaol [206] present in *Zingiber officinale*, menthol [207] present in *Mentha* × *piperita*. Recently, carvacrol, a constituent of *Origanum dictamnus* (Table 3), has also been confirmed to be an inhibitor not only of the Nav1.7 and Nav1.8 channels mentioned above, but also of Nav1.3 (localized in enterochromaffin cells, which detect mechanical and chemical stimuli in the intestine), Nav1.2 (currently no data supporting a role for Nav1.2 in pain or colonic sensory signaling) and Nav1.6 (having probably a primary role in transmitting rather than initiating action potentials) [208,209]. Probably Nav1.7 channel was also blocked by 1,8-cineole from *Achillea millefolium*, *Aloysia citriodora*, *Cinnamomum verum*, *Mentha* × *piperita*, *Peumus boldus*, *Rosmarinus officinalis* and *Salvia officinalis* (Table 3) in a patch-clamp experiment with rat superior cervical ganglia [210].

In humans Nav1.5 channels can be found in circular smooth muscles of jejunum and colon as well as in interstitial cells of Cajal. Their primary role is in mediating gastrointestinal motility and transit [208]. In a recent work using Nav1.5 a Nav1.7 CHO cells was confirmed, that protopine (from *Fumaria officinalis*) inhibits both types of mentioned channels [211]. Nav1.5 channel is also blocked by eugenol, as confirmed by an experiment with rat tissue [212] and may therefore contribute to the therapeutic effect of preparations of *Cinnamomum verum* (Table 3). 

### 5.9. Chloride Channels

TMEM16A (ANO1) is a calcium-activated chloride channel, which is found in primary sensory neurons and epithelial cells [213]. It is part of nociception processes [214], intestinal peristalsis [215] or mucin secretion [216].

It was found that liquiritigenin from *Glycyrrhiza glabra*, *Glycyrrhiza inflata*, and *Glycyrrhiza uralensis* (Table 3) inhibited human TMEM16A, which could explain its analgesic effect [217].

### 5.10. Adrenergic Receptors

The importance of GHR for the proper functioning of the GIT has already been emphasized several times in the previous text. This is confirmed by the findings of the α_2_-adrenergic receptor study. By deactivating or suppressing their expression, the level or activity of GHR is preserved [111,218]. 

The confirmed α_2_-adrenergic receptor antagonists in animal models are 10-gingerol, 6-shogaol, and 8-shogaol or glycycoumarin [219] from *Zingiber officinale* or from *Glycyrrhiza glabra*, *Glycyrrhiza inflata* and *Glycyrrhiza uralensis*, as well as curcumin from *Curcuma longa* and *Curcuma zanthorrhiza* [220].

### 5.11. Mucin Production

Stress also negatively affects mucus production and contributes to undesirable changes in the intestinal microflora [221]. The major building blocks in mucus are highly glycosylated proteins, mucins, excreted by goblet cells. 

The ability of hesperidin (orally twice 3 mg/kg/day) to increase mucus production in 80% acetic acid induced-gastric ulcer in rats was confirmed by the staining for the mucin-like glycoproteins method, the effect was more pronounced than with omeprazole (20 mg/kg under the same conditions) [222]. Hesperidin is a constituent of two plants in Table 3—*Gentiana lutea* and *Rosmarinus officinalis*. The same histochemical technique was used to confirm a 248% increase in gastric mucin production in mice after the oral application of the main constituent of *Peumus boldus*, boldine (100 mg/kg 1 h before administration of a mixture of 60% ethanol/0.3 M HCl (5 mL/kg, p.o.). Standard carbenoxolone (200 mg/kg) was not effective [223]. Ursolic acid (0.3 mg/kg 1 h before administration of a mixture of 60% ethanol/0.3 M HCl (5 mL/kg, p.o.), which is present in several plants from Table 3 (*Melissa officinalis*, *Mentha* × *piperita*, *Origanum dictamnus*, *Rosmarinus officinalis* and *Salvia officinalis*), reduced the size of gastric lesions in the mouse model by 83.11% compared to control. Carbenoxolone (200 mg/kg) was 96.55% effective [224].

In conclusion, one can see that, in the case of dyspepsia therapy, the mechanisms of action of the above-mentioned plant metabolites fit with the mechanisms of action of synthetic drugs.

## 6. Herbal Drugs Used in Functional Diarrhoea

The pathophysiology of functional diarrhoea is not sufficiently studied and includes various factors: altered GI motility, gut–brain disturbances, genetic and environmental factors, prior infections, and psychological factors [225]. Two synthetic drugs for diarrhoea are currently registered in the EU [15]. Loperamide is used as an antipropulsive [14]. Racecadotril decreases the intestinal hypersecretion of water and electrolytes [15]. Alternatively, the selective 5-HT_3_ receptor antagonist ondasetron (an antiemetic) is used, improving stool consistency, decreasing the frequency, and reducing the urgency of defecation [226]. Herbal medicines used for diarrhoea treatment represent a small indication group according to the number of EU monographs (Table 4). This is related to the small number of constituent types that should be responsible for the therapeutic efficacy of the related herbal medicines.

The extracts of the plants mentioned above exhibit antidiarrhoeal activity through one or more of the following effects: anti-secretory activity, enhanced intestinal absorption, anti-motility or anti-peristaltic effect, and anti-spasmodic action [227].

The antidiarrhoeal activity of tannins has long been attributed to the astringent effect on mucosal proteins, which leads to the formation of a protective layer. This reaction of tannins is possible due to their phenolic groups, which are able to form stable hydrogen bonds with proteins. Precipitated proteins of the gastrointestinal mucosa create a protective layer on the epithelial surface that would hamper the exudation of water, and then normalize intestinal hyperperistalsis. *Tri*-*O*-galloyl-β-d-glucose and *penta*-*O*-galloyl-β-d-glucopyranose were tested on the isolated colon of guinea pigs in a model of experimental diarrhoea with water secretion stimulated by rhein perfusion. Both tested tannins in a concentration of 0.1% completely inhibited the secretory effect of rhein, a natural laxative [228]. Galloylglucose derivatives are also found in *Rubus idaeus* and *Potentilla erecta* (Table 4). A large number of phenolic groups are also present in the condensed tannins in *Agrimonia eupatoria*, *Cinnamomum verum*, *Fragaria vesca*, *Fragaria moschata*, *Fragaria viridis*, *Fragaria × ananassa*, *Quercus robur*, *Quercus petraea*, *Quercus pubescens*, *Potentilla erecta* and *Vaccinium myrtillus* (Table 4) which may explain their antidiarrhoeal activity [229].

Current prospective approaches in the herbal treatment of (secretory) diarrhoea may include the use of inhibitors of (a) TMEM16A (calcium-activated chloride channel expressed in the smooth muscle of the gastrointestinal tract); (b) CFTR (cAMP-activated chloride channel); or (c) the KCNQ (Kv7) potassium channels—expressed in the epithelia of the intestine, both, or alternatively, PPARγ activators. 

Eugenol, a constituent of *Cinnamomum verum* (Table 4), was confirmed to have an inhibitory effect on TMEM16A and produced strong inhibition of intestinal contraction in mouse ileal segments in FRT cells (poorly differentiated epithelial cell line derived from Fischer rat thyroid gland) stably expressing human TMEM16A model and in interstitial cells of Cajal (Table 4) [230]. The effect of eugenol on this chloride channel was also confirmed in rat isolated ileum precontracted with KCl [231]. 

Moreover, tannins containing a galloyl or digalloyl residue in the molecule significantly inhibited in vitro Ca-activated chloride channel TMEM16A in human T84 colonic epithelial cells. At a concentration of 25 μM had *penta*-1,2,3,4,6-*O*-digalloyl-β-d-glucose IC_50_ = 3.1 μM, its derivatives, *penta*-1,2,3,4,6-*O*-galloyl-β-d-glucose and *di*-1,6-*O*-galloyl-β-d-glucose possess IC_50_ = 15 μM resp. 14 μM. Epicatechin gallate and epigallocatechin gallate were also active. On the other hand, the effect on CFTR and ENaC was very minimal. Thus, decreased TMEM16A-related chloride anion secretion in human intestinal cells provided a possible mechanism for the observed antidiarrhoeal properties of gallotannins [232], which are present in *Agrimonia eupatoria*, *Fragaria vesca*, *Fragaria moschata*, *Fragaria viridis*, *Fragaria × ananassa*, *Rubus idaeus* and *Potentilla erecta* (Table 4). TMEM16A inhibitors also include ubiquitous flavonoids luteolin, galangin, and quercetin [233].

The CFTR channel can be inhibited by condensed tannins, e.g., epicatechin, catechin, and procyanidin B2 [234], or by a hydrolyzable one, tannic acid [235].

A recently published study of the antidiarrhoeal action of *penta*-1,2,3,4,6-*O*-digalloyl-β-d-glucose (present in *Potentilla erecta*, Tabule 4) in a mouse model and with Chinese Hamster ovary cells suggests a link to inhibition of the KCNQ1/KCNE3 complex and an activation of KCNQ2/3 [236]. These mechanisms may also be potentiated by quercetin, an atypically acting KCNQ1 modulator present in plants [237].

The transcriptome analysis of the ileum in a mouse model identified that ellagic acid protect against castor oil-induced diarrhoea by activation the PPAR signaling pathway, mainly PPARγ [238]. Ellagic acid can be found in *Agrimonia eupatoria*, *Fragaria vesca*, *Fragaria moschata*, *Fragaria viridis*, *Fragaria × ananassa*, *Quercus robur*, *Quercus petraea*, *Quercus pubescens*, *Rubus idaeus*, and *Potentilla erecta* (Table 4).

Diarrhoea can also be a manifestation of a disturbed equilibrium function of aquaporins (AQPs) found in the small and large intestine. Depending on the triggering agent/model, diarrhoea is induced by a decrease [239,240] or, conversely, an increase [241,242] in AQP functionality or expression. There is not yet much knowledge about the antidiarrhoeal effects of natural compounds related to AQP. A gallotannin-rich extract from rhubarb significantly reduced the mRNA and protein expression levels of AQP2 and AQP3 in mucosal epithelial cells in the colons of diarrhoea mice and HT-29 cells, both induced by MgSO_4_ in a dose-dependent manner [243]. Gallotannins of analogous structure are contained in *Agrimonia eupatoria*, *Fragaria vesca*, *Fragaria moschata*, *Fragaria viridis*, *Fragaria × ananassa*, *Rubus idaeus*, and *Potentilla erecta* (Table 4).

In conclusion, mechanisms of action of the above-mentioned plants and synthetic drugs used in the therapy diarrhoea are similar.

## 7. Herbal Drugs Used in Functional Constipation

Constipation is a chronic disorder based on diverse etiologies with a reported prevalence ranging up to 27% in the general population [244]. The standard defecation process is dependent on 5-HT (serotonin), and balanced intestinal fluid/electrolytes regulation [245,246,247], thus FC can be treated by following drugs registered in EU [15]:

(1) prokinetic (prucalopride) acts as selective 5-HT_4_ agonist;
(2) osmotic laxatives (macrogol/polyethylene glycol, lactulose, sodium phosphate, sodium sulfate);
(3) producers of carbon dioxide (sodium bicarbonate + sodium dihydrogen phosphate) start a physical induction of reflex bowel evacuation;
(4) ionic surfactant (lauryl sulfate) decreases the surface tension at the stool oil–waterinterface, allowing water to penetrate the stool;
(5) synthetic stimulant laxatives (bisacodyl, dantron, sodium picosulfate, in fact their metabolite (bis-(*p*-hydroxyphenyl)-pyridyl-2-methane) open of L-type calcium channels, and BK channels, invoke Cl^−^ or HCO_3_^−^ secretion, and increase the prostaglandin release from macrophages which in turn decreases aquaporin expressions;
(6) secretagogue (lubiprostone), a ClC-2 chloride channels activator increases intestinal chloride secretion.

Herbal medicines used for constipation treatment represent a small indication group according to the number of EU monographs (Table 5).

Related to this is the small number of content types that are thought to be responsible for the therapeutic efficacy of the associated herbal medicines. Extracts from the plants mentioned above exhibit a laxative effect through one or more mechanisms.

### 7.1. Non-Digestible Polysaccharides

These polymeric molecules are found in *Linum usitatissimum*, *Plantago ovata*, *Plantago afra*, and *Plantago indica* (Table 5) and are often referred to as non-digestible fibers or ballast substances.

Their beneficial effect on intestinal peristalsis and defecation was described eighty years ago [248]. Subsequent human intervention studies have confirmed that non-digestible polysaccharides alleviate or eliminate constipation [249].

The mechanisms that determine the resulting effect are several and are determined by the chemical structure and physical properties of the polysaccharides. In the seeds of *Linum usitatissimum* (flax), polysaccharides with mucilage properties are found, which consist of two fractions. The acidic fraction (ca. 25%) is made up of galacturonic acid, rhamnose, galactose, and fucose, while the majority is made up of xylose, arabinose, and glucose. In addition to mucilage, flax seeds also contain pectin, which has rhamnose and galacturonic acid in the branched main chain, with galactose, xylose, and fucose bound to the chain. Seeds from *Plantago ovata*, *Plantago afra*, and *Plantago indica* contain branched arabinoxylans with different contents of the accompanying rhamnose and galactose [250]. Thus, all of the above plants contain arabinoxylans, which are not fermentable in vivo [251], but they are significantly bulk-forming [252].

It is this latter feature, in conjunction with mechanical irritation of the colonic wall, that has long been considered a sufficient explanation for the onset of defecation. The increase in intestinal motility, and thus the facilitation of defecation, may be related to the action of swollen polysaccharides on PIEZO2-type mechanoreceptors located in the intestinal epithelium, on enteroendocrine cells. These respond by releasing hormones and neurotransmitters, including 5-HT. Its increased level is associated with an increase in gut motility as shown in a mouse model [253,254]. It is now clear that this is only one of several and complementary mechanisms of the laxative effect of swelling non-digestible fibres.

The second group of polysaccharides, pectins (present in *Linum usitatissimum*), does not have a pronounced bulking ability but contributes to laxation by other mechanisms, the beginning of which is the fermentation of pectin by enterobacteria in the colon to form SCFAs. It is a clinically verified fact that constipation is accompanied by reduced mucin production in the intestine and its low lubricating capacity, which is thought to facilitate the movement of intestinal contents [255]. Previous observation that SCFAs acting in the rat intestine stimulate mucus production (with mucin being the main component) via a cholinergic nerve mechanism [256] has been confirmed and free fatty acid receptors 2 and 3 (FFAR2, FFAR3) have been proposed as the likely receptors that trigger the release of acetylcholine and the subsequent secretion of chloride anions [257]. Orthosteric agonists of these receptors are also SCFAs [258].

SCFAs also trigger 5-HT secretion from enterochromaffin cells in mice [259] by acting on FFA2 [260] and 5-HT is subsequently co-responsible for increased intestinal motility [254,261].

Recently, another possible mechanism of action of SCFAs in the treatment of constipation, voltage activation of the ClC-2 chloride channel, has been described [262].

### 7.2. Anthraglycosides

These constituents from *Aloe barbadensis*, *Aloe ferox*, *Rhamnus frangula*, *Rhamnus purshiana*, *Rheum palmatum* and *Senna alexandrina* (Table 5) are metabolised in the colon to laxative active aglycones [263]. Emodin (=frangula-emodin), by acting on mast cells, releases histamine from them, which binds to histamine H_1_ receptors on the intestinal epithelium, triggering an increase in chloride anion secretion. At the same time, histamine also binds to histamine H_2_ receptors of cholinergic neurons in the intestinal wall, releasing acetylcholine. This, by acting on muscarinic receptors of the intestinal epithelium also stimulates the secretion of chloride anions [264,265,266]. Emodin can also induce a laxative effect by increasing the water content of the colonic lumen by increasing AQP3 expression through activation of the pathway cAMP/PKA/p-CREB [267], respectively by decreasing the expression of AQP2 [268] or AQP4 [269].

However, it should be noted that the function of AQP3 in the intestinal epithelium is not unequivocally clear [270]. Its upregulation by the action of the well-established laxative, MgSO_4_, was observed in Caco-2 cells [271] also in rats [272], but after application of CuSO_4_ or HgCl_2_ to rats, despite the observed diarrhoea, no increase in AQP3 expression was observed, but even its inhibition [239]. Thus, the results of experiments with AQP3 depend on the experimental model used and the molecules tested. This is confirmed by the observed inhibition of its expression in Wistar rats after orally administered sennoside A [273], whereas, AQP3 expression was observed in Sprague-Dawley rats. In contrast, the expression of other aquaporins (AQP1, AQPs4–9) was inhibited. The active metabolite of sennoside A is thought to be reinanthrone, which activates macrophages to increased production of PGE_2_, which inhibits AQP3 expression in the colon, resulting in water retention and stool dilution there. Another metabolite, rhein, shows no such activity [274].

Recently, the positive effect of *Rheum palmatum* extract containing rhein has been confirmed in a mouse model of constipation by initiating the release of acetylcholine and histamine as well as by increasing butyric acid levels also on mucus production [275].

Rhein and aloe-emodin in a cell-based fluorescent assay activated the CFTR chloride channel [276,277], which leads to an increase in the secretion of chloride anions into the intestinal lumen and subsequently sodium cations, thereby increasing the secretion of intestinal fluid and diluting the intestinal contents.

Moreover, in the stripped descending colon from Wistar rat model, rhein inhibits Na^+^/K^+^-ATPase, which reduces the reabsorption of ions and water from the intestinal lumen [278].

The last here reported mechanism of antraglycoside laxatives has been elucidated for emodin and *Rheum palmatum* extract hydrolysate, which additionally contains chrysophanol, physcione, rhein, and aloe-emodin as aglycones [279]. In an in vitro model of isolated ICR mice intestine and subsequently by molecular docking, the inhibitory effect of the aforementioned aglycones on β_2_-adrenergic receptors was confirmed, which was manifested by accelerated colonic peristalsis.

### 7.3. Higher Fatty Acids

Official European phytotherapy lists three plants (Table 5) as sources of oil containing substances of this structural group. *Linum usitatissimum* seed oil contains mainly α-linolenic, oleic and linoleic acids, *Polypodium vulgare* rhizome oil contains palmitic, oleic, and linoleic ones, and *Ricinus communis* seed oil contains up to 92% ricinoleic acid [45].

Increased contractility of rat intestinal muscle after treatment with long chain fatty acids [280] has been linked to activation of the GPR40 receptor and subsequent upregulation of Ca^2+^ [281]. In healthy volunteers, oleic acid induced intestinal contraction and accelerated the transit of intestinal contents [282]. It is likely that PGE_2_ is involved in this effect in the rat model [283] and in the mouse model either PPARα [284,285] or PPARγ activation [285,286].

The best one characterized is ricinoleic acid. It inhibits Na^+^/K^+^-ATPase [287], decreases active absorption of electrolytes [288] by impairing the functionality of the intestinal mucosa [289], via eNOS activation [290], by the stimulation of chloride anion secretion in the rat colon via a prostaglandin-dependent and a partly neural mechanism, which may involve guanylate cyclase [291], and in a mouse model by the activation of intestinal smooth muscle via EP_3_ prostanoid receptors [292]. The originally assumed stimulatory effect of PGE_2_ on the rat intestine [283] is neither probable [292] nor excluded [293].

In conclusion, plant metabolites compared to synthetic drugs show more types of mechanisms of action potentially usable in diarrhoea treatment.

## 8. Herbal Drug Used in Nausea and Vomiting

The population prevalence of NVDs is approximately 2% [294]. Actually, in the EU, antiemetics include 5-HT_3A_ receptor antagonists (granisetron, ondansetron, palonosetron, and tropisetron, respectively), a H_1_ histamine receptor antagonist (dimenhydrinate), and an NK-1 (substance P) receptor antagonist (aprepitant) [15]. However, cyclizine (a H_1_ antagonist with antimuscarinic activity at M_1_, M_2_ and M_3_ receptors), metoclopramide (a D_2_ and 5-HT_3A_ antagonist), and domperidone (a D_2_ and D_3_ antagonist) are also used in clinical practice [295].

Currently, only *Zingiber officinale* is allowed for official European phytotherapy of nausea and vomiting. Its antiemetic effect is related to a specific structural group, the arylalkanones (6-gingerol, 8-gingerol, 10-gingerol, their degradation products (6-shogaol and zingerone)) and the diterpene galanolactone. For all of these compounds, inhibition of 5-HT_3_ receptors, as discussed above (Section 5), has long been considered to be the crucial mechanism of antiemetic action. However, the antiemetic efficacy of ginger extracts has not always been clearly correlated with inhibition of 5-HT_3_ receptors in neural pathways, but may play a role in controlling the perception of the environment by the eyes [296].

Confirmation of the putative importance of cholinergic pathways in motion sickness [297] and the role of M_3_ receptors inhibition by gingerols and 6-shogaol was discovered later [298]. Recently, the complexity of the action of the components of ginger has been confirmed:

(a) 6-shogaol directly activates vagal afferent C-fibres of peripheral gastrointestinal endings, and there is evidence supporting the hypothesis that TRPA1 plays a critical role in mediating this activation [299]; TRPA1 activation has been described for 10-shogaol [134] and for a not specified gingerol [300];
(b) and not specified gingerol not only suppresses the production of 5-HT and the expression of its receptors, but also NK1 receptors, tryptophan hydroxylase, substance P and its precursor preprotachykinin, dopamine and its D_2_ receptor, in the intestine and in the area postrema of the rat and mink brainstem (in a model of cisplatin-induced vomiting), and also enhances the accumulation of dopamine and serotonin transporters, and of the neutral endopeptidase. The result is suppression of vomiting [107,301];
(c) activation of L-type calcium channels is known to cause vomiting, whereas blockade of these ion channels with nifedipine-like antagonists is antiemetic [302]. Therefore, it is likely that the inhibition of calcium channels induced by ginger extract [303] and by its component, 8-gingerol [304], is involved in an antiemetic effect of *Zingiber officinale*.

In conclusion, the constituents of ginger have only a partial overlap of their mechanisms of action with synthetic antiemetics.

## 9. Factors Influencing the Use of Herbal Medicines

It is typical for herbal medicine to contain tens to hundreds of constituents in mutually stable but not equivalent proportions, where individual constituents may exhibit the desired bioactivity of varying intensity, or may be inactive but positive/negative modifying cofactors, or may have no effect on the therapeutic activity at all. The resulting bioactivity is thus the sum of a myriad of sub-effects [305].

The possibility of using plants in the therapy of human diseases, including GIT diseases, is determined not only by the biological activity of their constituents, but also by their degradation products after the action of enterobacteria or biochemical processes of the human body. Exemplary examples are the antraglycosides or their aglycones discussed in Section 7. Anthraquinone aglycones of rhubarb have been confirmed to synergistically enhance the laxative effect in constipated rats by direct and indirect mechanisms simultaneously [306]. Explaining why flaxseed consumption is better than psyllium consumption for relieving constipation in *diabetes mellitus* type 2 patients with constipation [307], may be related to the more complex action of the two types of non-digestible polysaccharides of *Linum usitatissimum* (arabinoxylans and pectins) compared to the action of psyllium arabinoxylans alone (see Section 7).

Interestingly, butyrate arising from the metabolism of pectin affects mucin production in a biphasic manner: at low levels, below 5 mM, it is stimulatory, above that level inhibitory [149]. This opens up a discussion on the effective and beneficial level of active molecules, which is related to their bioavailability. The latter is generally estimated to be ca. 1%, so that e.g., polyphenols (flavonoids, catechins, phenolic acids) in blood plasma reach in vivo levels of a few μM [308]. Although such values are usually considered insufficient to produce the desired pharmacological effect, this may not be the case. The M_3_ receptor or β_2_-adrenoceptors show sensitivity to many ligands already at sub-picomolar concentrations in in vitro or ex vivo models [309], thus, it is not surprising that caffeic acid exerts a positive effect via modulation of the intracellular redox state on glucose-induced endothelial dysfunction already at an in vitro concentration of 10 nM [310] or anthocyanins activate membrane estrogen receptors, and subsequently induce the vasodilation of rat mesenteric aorta ex vivo at a concentration of 100 nM [311].

An equally important issue with the use of over-the-counter herbal medicines is their potential for drug–drug interactions. When taking another drug/drugs (even synthetic ones) simultaneously for a different health problem than the one addressed by the “newly added” herbal remedy; knowledge of the pharmacodynamic (as well as pharmacokinetic) action of the active ingredients of all drugs acting in the body is a prerequisite for minimizing the risk of drug–drug interactions. As an example, the possible interactions of tizanidinium chloride (an α_2_-agonist used for the short-term treatment of muscle spasticity) with drugs from *Zingiber officinale* containing α_2_-antagonists 10-gingerol, 6-shogaol, and 8-shogaol have not been reported anywhere (not yet observed) [219] or the interaction of ivacaftor (a CFTR potentiator used to treat the management of cystic fibrosis) with tannin-containing preparations (CFTR inhibitors) used in the treatment of diarrhoea [234,235].

## 10. Conclusions

Traditional phytotherapy has a history of many thousands of years and its empirical knowledge has been the basis of official practices in recent decades, which are now regulated by legislation in the EU [5]. This directive requires at least a basic knowledge of the mechanism of action of herbal medicines only in the category of “well-established medicinal use”, which in the case of the treatment of GIT diseases, is only a small group of herbal laxatives—the exception being *Polypodium vulgare* (Section 7) and a single plant from the field of herbal antiemetics, *Zingiber officinale* (Section 8). The remaining areas—heartburn (Section 4), mild dyspeptic/gastrointestinal disorders including symptoms of temporary loss of appetite (Section 5) and diarrhoea (Section 6)—contain a number of plants in the “traditional use” category, only (see Section 3). For this category, knowledge of the mechanism of action is not required to be known and reported in related documents, although the quantity and quality of such information have increased enormously in recent decades.

The use of information on the cellular and molecular targets of plant metabolites is important not only for the research and development of effective phytomedicines for a given medical complication but also to ensure the safety of such therapies in the context of avoiding drug-drug interactions. Of course, the clinical relevance of possible drug interactions cannot be guaranteed from in vitro data, but any (theoretical) knowledge can prevent a possible negative outcome of the use of herbal medicines and thus ultimately enhance/preserve their reputation not only in the eyes of experts (physicians and pharmacists) but also to the general public. Another benefit of knowledge of the mechanism of action of plant metabolites may also be the development of new multi-component herbal medicines in which their components will harmoniously complement each other by different actions on the desired targets.

In conclusion, this review summarizes the current knowledge on the mechanism of action of medicinal plant constituents used in official European phytotherapy and opens a discussion on their possible use in justified cases as an equivalent substitute for synthetic preparations based on identical cellular and molecular mechanisms.

## Figures and Tables

**Table 1:** In vitro H^+^/K^+^-ATPase inhibitory activity of plant secondary metabolites.

Metabolite	IC_50_ (M)	Model
verbascoside	9.7 × 10^−5^	gastric microsomes isolated from rat stomach [20]
omeprazole (control)	8.75 × 10^−5^	
catechin	1.7 × 10^−4^	enzyme from pig gastric mucosa [21]
(−)-epicatechin	4.7 × 10^−5^	
(−)-epigallocatechin	9.3 × 10^−5^	
(−)-epicatechin gallate	1.1 × 10^−7^	
(−)-epigallocatechin gallate	6.9 × 10^−9^	
cyanidin	1.0 × 10^−6^	enzyme from pig gastric mucosa [22]
delphinidin	2.9 × 10^−6^	
quercetin	3.4 × 10^−6^	
quercetagetin	4.0 × 10^−6^	
luteolin	5.5 × 10^−6^	
eriodictyol	6.9 × 10^−6^	
pelargonidin	7.0 × 10^−6^	
kaempferol	7.4 × 10^−6^	
quercetin-3-glucoside	9.5 × 10^−6^	
quercetin-3-rhamnoside	1.7 × 10^−5^	
quercetin-3-galactoside	2.0 × 10^−5^	
luteolin-7-glucoside	3.0 × 10^−5^	
taxifolin	4.4 × 10^−5^	
quercetin-3-gluco-rhamnoside (rutin)	1.0 × 10^−4^	
tannic acid	2.9 × 10^−8^	enzyme from pig gastric mucosa [23]

**Table 2:** Herbal drugs used for temporary loss of appetite treatment [43].

Plant ^a^	Plant Part, Herbal Drug ^a^	Main Constituents ^b,c^
*Achillea millefolium* L.	flower, Millefolii flos aerial part, Millefolii herba	**sesquiterpenic lactones, essential oil, chlorogenic acid**, flavonoids
*Artemisia absinthium* L.	aerial part, Absinthii herba	**sesquiterpenic lactones, essential oil, chlorogenic acid**, flavonoids
*Arctium lappa* L. (=*Arctium major* Gaertn.) *Arctium minus* (Hill) Bernh. *Arctium tomentosum* Mill.	root, Arctii radix	**cynarine, chlorogenic acid**, polysaccharides, triterpenes, lignans
*Centaurium erythraea* Rafn *s*. *l*. *Centaurium majus* (H. et L.) Zeltner *Centaurium suffruticosum* (Griseb.) Ronn.	aerial part, Centaurii herba	**secoiridoids**, flavonoids, xanthones
*Cetraria islandica* (L.) Acharius *s*. *l*.	thallus, Lichen islandicus	**lichenic acids**, polysaccharides, unsaturated aliphatic acids
*Cichorium intybus* L.	root, Cichorii radix	**sesquiterpenic lactones**, inulin, polyines
*Gentiana lutea* L.	root, Gentianae radix	**secoiridoids, gentiobiose, gentianose**, essential oil, xanthones
*Harpagophytum procumbens* DC. *Harpagophytum zeyheri* Decne.	root, Harpagophyti radix	**iridoids**, phenylethanoids, flavonoids
*Marrubium vulgare* L.	aerial part, Marrubii herba	**diterpenes**, flavonoids, betonicine
*Menyanthes trifoliata* L.	leaf, Menyanthidis trifoliatae folium	**iridoids**, flavonoids, phenolic acids
*Taraxacum officinale* F.H. Wigg.	aerial part + root, Taraxaci officinalis herba cum radice	**sesquiterpenic lactones, chlorogenic acid**, inulin, flavonoids
*Trigonella foenum-graecum* L.	seed, Trigonellae foenugraeci semen	**steroidal saponins, trigonelline**, flavonoids, stilbenes, mucilage

^a^ a botanical terminology as used in European Pharmacopoeia 10.8. [44]; ^b^ main constituents by Nagy et al. [45]; ^c^ bitter constituents in bold.

**Table 3:** Herbal drugs and related essential oils used for mild dyspepsia treatment [47].

Plant ^a^	Plant Part, Herbal Drug ^a^	Main Constituents ^b^
*Achillea millefolium* L.	flower, Millefolii flos aerial part, Millefolii herba	sesquiterpenic lactones, essential oil, chlorogenic acid, flavonoids
*Aloysia citriodora* Paláu	leaf, Verbenae citriodorae folium	essential oil, flavonoids, verbascoside
*Althaea officinalis* L.	root, Althaeae radix	mucilage, flavonoids, cinnamic acid derivatives
*Artemisia absinthium* L.	aerial part, Absinthii herba	sesquiterpenic lactones, essential oil, chlorogenic acid, flavonoids
*Carum carvi* L.	fruit, Carvi fructus	essential oil, lignans, coumarins
	essential oil, Carvi aetheroleum	various essential oil compounds
*Centaurium erythraea* Rafn *s*. *l*. *Centaurium majus* (H. et L.) Zeltner *Centaurium suffruticosum* (Griseb.) Ronn.	aerial part, Centaurii herba	secoiridoids, flavonoids, xanthones, cinnamic acid derivatives
*Chamaemelum nobile* (L.) All.	flower, Chamomillae romanae flos	sesquiterpenic lactones, essential oil, flavonoids
*Cichorium intybus* L.	root, Cichorii radix	sesquiterpenic lactones, inulin, polyines
*Curcuma longa* L.	rhizome, Curcumae longae rhizoma	curcuminoids, essential oil, starch
*Curcuma zanthorrhiza* Roxb.	rhizome, Curcuma zanthorrhizae rhizoma	curcuminoids, essential oil, flavonoids, starch
*Cinnamomum verum* J. Presl.	bark, Cinnamomi cortex	essential oil, proanthocyanidins, cinnamic acid derivatives
	essential oil, Cinnamomi corticis aetheroleum	various essential oil compounds
*Cynara cardunculus* L. *Cynara scolymus* L.	leaf, Cynarae folium	sesquiterpenic lactones and their glycosides, cynarine, chlorogenic acid, flavonoids
*Foeniculum vulgareMiller* ssp. *vulgare* var. *vulgare*	fruit, Foeniculi amari fructus	essential oil, glucosides of monoterpenoids, oil
*Foeniculum vulgareMiller* ssp. *vulgare* var. *dulce*	fruit, Foeniculi dulcis fructus	essential oil, glucosides of monoterpenoids, oil
*Gentiana lutea* L.	root, Gentianae radix	secoiridoids, flavonoids, gentiobiose, gentianose, essential oil, xanthones
*Glycyrrhiza glabra* L. *Glycyrrhiza inflata* Bat. *Glycyrrhiza uralensis* Fisch.	root, Liquiritiae radix	saponins, flavonoids, coumarins
*Harpagophytum procumbens* DC. *Harpagophytum zeyheri* Decne.	root, Harpagophyti radix	iridoids, phenylethanoids, flavonoids
*Helichrysum arenarium* (L.) Moench.	flower, Helichrysi flos	flavonoids, coumarins, chlorogenic acid, essential oil
*Hypericum perforatum* L.	aerial part, Hyperici herba	phloroglucinols, naphtodianthrones, flavonoids, tannins, essential oil
*Juniperus communis* L.	fleshy cone, Juniperi galbulus essential oil, Juniperi aetheroleum	essential oil, flavonoids, diterpenes various essential oil compounds
*Linum usitatissimum* L.	seed, Lini semen	mucilage, lignans, cyanogenic glycosides
*Malva neglecta* Wallr. *Malva sylvestris* L.	leaf, Malvae folium	mucilage, flavonoids
*Malva sylvestris* L.	flower, Malvae sylvestris flos	mucilage, flavonoids, anthocyanins
*Marrubium vulgare* L.	aerial part, Marrubii herba	diterpenes, flavonoids, betonicine
*Matricaria chamomilla* L.	flower, Matricariae flos	essential oil, flavonoids, coumarins, polysaccharides
*Melissa officinalis* L.	leaf, Melissae folium	essential oil, flavonoids, chlorogenic acid, triterpenes
*Mentha* × *piperita* L.	leaf, Menthae piperitae folium	essential oil, flavonoids, cinnamic acids derivatives, triterpenes
	essential oil, Menthae piperitae aetheroleum	various essential oil compounds
*Menyanthes trifoliata* L.	leaf, Menyanthidis trifoliatae folium	iridoids, flavonoids, phenolic acids
*Origanum dictamnus* L.	aerial part, Origani dictamni herba	essential oil, flavonoids, triterpenes
*Origanum majorana* L.	aerial part, Origani majoranae herba	essential oil, flavonoids, chlorogenic acid
*Peumus boldus* Molina.	leaf, Boldi folium	alkaloids, essential oil, flavonoids
*Pimpinella anisum* L.	fruit, Anisi fructus essential oil, Anisi aetheroleum	essential oil, flavonoids, coumarins various essential oil compounds
*Pistacia lentiscus* L.	oleoresin, Mastix	essential oil, triterpenes
*Rosmarinus officinalis* L.	leaf, Rosmarini folium	essential oil, flavonoids, diterpenes, triterpenes
	essential oil, Rosmarini aetheroleum	various essential oil compounds
*Salvia officinalis* L.	leaf, Salviae officinalis folium	essential oil, flavonoids, cinnamic acid derivatives, diterpenes
*Sideritis* sp.	aerial part, Sideritis herba	essential oil, flavonoids, verbascoside, diterpenes
*Silybum marianum* L.	fruit, Silybi mariani fructus	flavonoligans, flavonoids, unsaturated fatty acids
*Taraxacum officinale* F.H. Wigg.	aerial part + root, Taraxaci officinalis herba cum radice	sesquiterpenic lactones, chlorogenic acid, inulin, flavonoids
*Zingiber officinale* Roscoe	rhizome, Zingiberis rhizoma	essential oil, arylalkanones

^a^ a botanical terminology as used in European Pharmacopoeia 10.8. [44]; ^b^ main constituents by Nagy et al. [45].

**Table 4:** Herbal drugs used for functional diarrhoea [47].

Plant ^a^	Plant Part, Herbal Drug ^a^	Main Constituents ^b^
*Agrimonia eupatoria* L.	aerial part, Agrimoniae herba	tannins, flavonoids, triterpenes, polysaccharides
*Cinnamomum verum* J. Presl.	bark, Cinnamomi cortex	essential oil, proanthocyanidins, cinnamic acid derivatives, polysaccharides
*Fragaria vesca* L. *Fragaria moschata* Weston *Fragaria viridis* Weston *Fragaria* × *ananassa* (Weston) Duchesne ex Rozier	leaf, Fragariae folium	tannins, flavonoids, cinnamic acid derivatives
*Quercus robur* L. *Quercus petraea* (Matt.) Liebl. *Quercus pubescens* Willd.	bark, Quercus cortex	tannins, triterpenes
*Rubus idaeus* L.	leaf, Rubi idaei folium	tannins, flavonoids, cinnamic acid derivatives, benzoic acid derivatives
*Potentilla erecta* (L.) Raeusch.	rhizome, Tormentillae rhizoma	tannins, triterpenes, flavonoids
*Vaccinium myrtillus* L.	fruit, Myrtilli fructus siccus	tannins, anthocyanins, flavonoids, cinnamic acid derivatives, pectin

^a^ a botanical terminology as used in European Pharmacopoeia 10.8. [44]; ^b^ main constituents by Nagy et al. [45].

**Table 5:** Herbal drugs used in functional constipation [47].

Plant ^a^	Plant Part, Herbal Drug ^a^	Main Constituents ^b^
*Aloe barbadensis* Miller *Aloe ferox* Mill. and its hybrids	dried juice of leaves, Aloes folii succus siccatus	aloin A and B, glycosylchromones aloin A and B, aloinoside A and B
*Linum usitatissimum* L.	seed, Lini semen	polysaccharides, oil, lignans, cyanogenic glycosides
*Plantago ovata* Forssk.	seed, Plantaginis ovatae semen outer integument of a seed, Plantaginis ovatae seminis tegumentum	polysaccharides, oligosaccharides, sterols, proteins
*Plantago afra* L. *Plantago indica* L.	seed, Psyllii semen	polysaccharides, sterols, proteins
*Polypodium vulgare* L.	rhizome, Polypodii rhizoma	steroidal saponins, steroids, triterpenes, oil
*Ricinus communis* L.	refined oil, Ricini oleum raffinatum virgin oil /Ricini oleum virginale	ricinoleic acid, linoleic acid, oleic acid
*Rhamnus frangula* L.	bark, Frangulae cortex	glucofrangulin A and B, frangulin A and B, frangula-emodin
*Rhamnus purshiana* DC.	bark, Rhamni purshianae cortex	cascarosides A—F, aloin A and B
*Rheum palmatum* L.	root, Rhei radix	mono- and diglucosides of: rein, aloe- emodin, frangula-emodin, chysophanol
*Senna alexandrina* Mill.	fruit, Sennae fruit	sennosides A—D, palmidines A—D, rheidines A—C, tannins
	leaflets, Sennae foliolum	sennosides A—D

^a^ a botanical terminology as used in European Pharmacopoeia 10.8. [44]; ^b^ main constituents by Nagy et al. [45].

## Data Availability

All tables are created by the authors. All sources of information are adequately referenced. There is not need to obtain copyright permissions.

## Supplementary Materials

The following supporting information can be downloaded at: https://www.mdpi.com/article/10.3390/molecules27092881/s1, Table S1: Abbreviations. Table S2: List of constituents mentioned in the manuscript and their activities.
