# Supplementary material for: Treatment of Gastrointestinal Disorders—Plants and Potential Mechanisms of Action of Their Constituents
Source: Molecules. 2022 Apr 30;27(9):2881. doi: 10.3390/molecules27092881 (PMC9105531; doi:10.3390/molecules27092881)
Supplement: Supplementary file 1 [file molecules-27-02881-s001.zip › molecules-1666235-supplementary.pdf]

molecules

MDPI

Supplementary Materials

Treatment of Gastrointestinal Disorders—Plants and Potential Mechanisms of Action of their Constituents

Szilvia Czigle *, Silvia Bittner Fialová, Jaroslav Tóth, Pavel Mučaji, Milan Nagy and on behalf of the OEMONOM †

Department of Pharmacognosy and Botany, Faculty of Pharmacy, Comenius University Bratislava, Odbojárov 10, SK-832 32 Bratislava, Slovakia; silvia.bittner.fialova@uniba.sk (S.B.F.); jaroslav.toth@uniba.sk (J.T.); mucaji@fpharm.uniba.sk (P.M.); nagy@fpharm.uniba.sk (M.N.)
* Correspondence: szilvia.czigle@uniba.sk; Tel.: +421250117209
† Listed at the end of Acknowledgments.

Table S1. Abbreviations.

**Abbreviations**
A2 receptor – adenosine 2 receptor
A7r5 cells – rat thoratic aorta smooth muscle cell line
AGHR – acyl ghrelin
AQPs1–9 – aquaporins 1–9
ATC system – Anatomical Therapeutic Chemical Classification System
α_2-adrenergic receptor – alfa 2-adrenergic receptor
β_2-adrenergic receptor – beta 2-adrenergic receptor
BK (= KCa1.1 = KCNMA1) channel – big potassium channel, calcium-activated potassium channel subfamily M alpha 1
C57BL/6N – inbred strain of laboratory mouse
CA3–CA1 – CA1–CA4 regions of the cornu Ammonis (CA), hippocampus
Caco-2 cells – cell line derived from a colorectal adenocarcinoma patient
CALHM1 – calcium homeostasis modulator protein 1
cAMP – cyclic adenosine monophosphate
CCK – cholecystokinin
C-fibers – group C nerve fibers
CFTR – cystic fibrosis transmembrane conductance regulator protein
cGMP – cyclic guanosine monophosphate
ClC-2 – chloride channel 2
CNS – central nervous system
COX-1 – cyclooxygenase 1
CYP 450 – cytochrome P450
D_1, D_2, D_3 receptor – dopamine receptor D_1, D_2, D_3
DBGI – Disorders of Brain-Gut Interactions
DRG – dorsal root ganglion
EMA – European Medicines Agency
ENaC – epithelial sodium channel
eNOS – endothelial nitric oxide synthase
ENS – enteric nervous system
EP_3 receptor – prostaglandin EP_3 receptor
EU – European Union
FC – functional constipation
FDI – functional diarrhoea
FDY – functional dyspepsia

FFAR2, FFAR3 – free fatty acid receptors 2 and 3
FGIDs – functional gastrointestinal disorders
FH – functional heartburn
FRT cells – Fischer rat thyroid epithelial cell line
G cells – gastrin cells
GAS – gastrin
GHR – ghrelin
GI – gastrointestinal
GIT – gastrointestinal tract
GLP-1 – glucagon-like peptide-1
GPR40 receptor – G protein-coupled receptor 40
H_1, H_2 receptors – histamine receptors H_1, H_2
HEK293, HEK tsA201 cells – human embryonic kidney cell line
HET-1A cells – normal oesophageal epithelium cell line
HGT-1 cells – human gastric cancer cell line
HMPC – Committee on Herbal Medicinal Products
5-HT – 5-hydroxytryptamine (serotonin)
hTRPV1 – human transient receptor potential channel (TRPV1)
HT-29 cells – human colorectal adenocarcinoma cell line
5-HT_{2C}, 5-HT_3, 5-HT_{3A}, 5-HT_4 receptors
– 5-hydroxytryptamine (serotonin) receptors 5-HT_{2C}, 5-HT_3, 5-HT_{3A}, 5-HT_4 subtypes
IC_{50} (M) – inhibitory concentration 50% (molar concentration)
ICAM-1 – intercellular adhesion molecule 1
ICCs – interstitial cells of Cajal
ICR – Institute of Cancer Research
IFN-γ – interferon gamma
IgA, IgG, IgM – immunoglobulins A, G, M
IL-1β, IL-2, IL-6, IL-8, IL-12 – interleukins 1β, 2, 6, 8, 12
iNOS – inducible nitric oxide synthase
IP_3 – inositol triphosphate
IP_3R – inositol triphosphate receptor
K^+-ATPase – ATP-sensitive potassium channel
K2P2.1 (KCNK2, TREK-1), K2P4.1 (KCNK4, TRAAK), K2P10.1 (KCNK10, TREK-2), K2P18.1 (KCNK18, TWIK) – potassium channels (potassium channel subfamilies K member 2, 4, 10, 18)
K_{ATP} cascade – ATP-sensitive potassium channel cascade
KCNQ (KCNQ1, KCNQ2, KCNQ3) – potassium voltage-gated channels subfamily Q member 1, 2, 3
KCNQ1/KCNE3 complex – complex of potassium voltage-gated channel subfamily Q member 1 and potassium voltage-gated channel subfamily E regulatory subunit 3
Kv7 – voltage-gated potassium channels family Kv7
LCFA – long chain fatty acid
LOX-5 – lipoxygenase 5
LTB4 – leukotriene 4
M_1, M_2 and M_3 receptors (mAChRs) – muscarinic acetylcholine receptors M_1, M_2 and M_3
MOT – motilin
mRNA – messenger ribonucleic acid
nAChRs (α3β4 nAChRs, α4β2 nAChRs, α7 nAChRs) – nicotinic acetylcholine receptors type α3β4, α4β2, α7
Nav1.2, Nav1.3, Nav1.5, Nav1.6, Nav1.7 channels – voltage-gated sodium channels 1.2, 1.3, 1.5, 1.6, 1.7

NF-κB – nuclear factor-kappa B
NICE – The National Institute for Health and Care Excellence
NK-1 receptor – neurokinin-1 receptor
NVDs – nausea and vomiting disorders
OATP – organic-anion-transporting polypeptide
p-CREB – phosphorylated cAMP-responsive element-binding protein
PGE_2 – prostaglandin E_2
P-gp – P-glycoprotein, permeability glycoprotein
PIEZO2-type mechanoreceptor
– piezo type mechanosensitive ion channel component 2
PKA – cAMP-protein kinase A
PLA2 – phospholipases A2
PLCβ2 – phospholipase C β2
PPAR (PPARα, PPARγ) – peroxisome proliferator-activated receptors α, γ
PPIs (H^+/K^+-ATPase inhibitors) – proton pump inhibitors
PYY – peptide YY
SCFA – short-chain fatty acid
SSRI – selective serotonin reuptake inhibitor
T84 cells – human colonic adenocarcinoma cell line
TAS2R – bitter taste receptor
T-cell – type of lymphocyte
TG – trigeminal ganglion
TMEM16A (ANO1) – transmembrane 16 (Anoctamin 1) protein mediates calcium-activated chloride channel (CaCC)
TNF-α – tumour necrosis factor alpha
TRP channels (superfamilies TRPA1, TRPC1, TRPC5, TRPM5, TRPM7, TRPM8, TRPV1) – transient receptor potential (TRP) channels
TU – traditional use
TXB_2 – thromboxane B_2
VGNC – voltage-gated sodium channel
WEU – well established use

Table S2. List of constituents mentioned in the manuscript and their activities.

Constituents	Test	Activities	References
aloe-emodin	in vivo	CFTR channel activation	[277]
	ex vivo	β_2-adrenergic receptor inhibitor	[279]
anethole	in vitro	TRPA1 activator	[174]
anisaldehyde	in vitro	TRPA1 activator	[173]
anthraglycosides	in vivo	variable action on AQPs expression	[268–275]
arylalkanones	in vitro	anti-inflammatory activity	[45]
	in vivo	anti-inflammatory activity	[45]
	human	anti-inflammatory activity	[45]
biochanin A	in vitro	increase of gastric acid production through PPARγ activation	[69]
(−)-α-bisabolol	in vitro	TRPA1 activator	[167]
boldine	in vitro	negative allosteric modulator of 5-HT_3 receptor	[99]
	in vivo	mucus production increase	[223]
borneol	in vitro	TRPM8 agonist	[187]
caffeine	in vitro	increase of gastric acid production through TAS2R	[64]
	in vitro	increase of gastric acid production through A2 receptor antagonism	[66]
camphor	in vitro	TRPV1 biphasic agonist	[137]
	in vitro	TRPA1 activator	[176]
	in vitro	TRPM8 agonist	[186]
capsaicin	human	TRPV1 biphasic agonist	[122]
carnosic acid	in vitro	TRPA1 activator	[178]
carnosol	in vivo	decrease in the gastric lesion index	[18]
	in vitro	TRPA1 activator	[178]
carvacrol	in vitro	TRPA1 activator	[165]
	in vitro	TRPM7 inhibitor	[171]
	in vitro	K2P2.1 activator	[199]
	in vitro	Nav1.2, Nav1.3, Nav1.6, Nav1.7, and Nav1.8 inhibitor	[209]
carvone	in vitro	TRPV1 biphasic agonist	[137]
catechin	in vitro	H^+/K^+-ATPase inhibitor	[21]
	in vitro	CFTR channel inhibitor	[234]
1,4-cineole	in vitro	TRPA1 activator	[166]
	in vitro	TRPM8 agonist	[166]
1,8-cineole	in vitro	negative allosteric modulator of 5-HT_3 receptor	[97]
	in vitro	TRPV1 biphasic agonist	[137]
	in vitro	TRPM8 agonist	[166]
	in vitro	? Nav1.7 inhibitor	[210]
cinnamaldehyde	in vitro	TRPA1 agonist	[161]
	in vitro	K2P2.1 activator	[199]
citral	in vitro	TRPV1 biphasic agonist	[137]

	in vitro	TRPA1 activator	[175]
citronellal	in vivo	inhibitor of $\alpha3\beta4$ nAChR	[82]
	in vitro	TRPV1 biphasic agonist	[137]
citronellol	in vitro	negative allosteric modulator of 5-HT_3 receptor	[97]
	in vitro	K2P2.1 activator	[199]
p-cumenol	in vitro	K2P2.1 activator	[199]
curcumin	in vivo	TRPV1 antagonist	[142]
	ex vivo	TRPV1 antagonist	[142]
	in vitro	TRPM8 inactive (human)	[188]
	in vitro	TRPM8 antagonist (rat)	[163]
	in vitro	KCa1.1 activator	[202]
	in vivo	α_2-adrenergic receptor antagonist	[220]
cyanidin	in vitro	H^+/K^+-ATPase inhibitor	[22]
p-cymene	in vitro	K2P2.1 activator	[199]
cynaropicrin	ex vivo	competitive antagonist of AChR	[84]
delphinidin	in vitro	H^+/K^+-ATPase inhibitor	[22]
4,5-dicaffeoylquinic acid	in vitro	TRPV1 biphasic agonist	[137]
di-1,6-*O*-galloyl-β-D-glucose	in vitro	TMEM16A inhibitor	[232]
ellagic acid	in vitro	PPARγ activator	[238]
emodin	in vivo	histamine release from mast cells (H_1 receptor, H_2 receptor)	[264–266]
	ex vivo	β_2-adrenergic receptor inhibitor	[279]
(−)-epicatechin	in vitro	H^+/K^+-ATPase inhibitor	[21]
	in vitro	CFTR channel inhibitor	[234]
(−)-epicatechin gallate	in vitro	H^+/K^+-ATPase inhibitor	[21]
	in vitro	TMEM16A inhibitor	[232]
(−)-epigallocatechin	in vitro	H^+/K^+-ATPase inhibitor	[21]
(−)-epigallocatechin gallate	in vitro	TMEM16A inhibitor	[232]
eriodictyol	in vitro	H^+/K^+-ATPase inhibitor	[22]
	in vitro	TRPV1 antagonist	[137]
essential oils	in vitro	anti-inflammatory activity	[45]
	in vivo	anti-inflammatory activity	[45]
	human	anti-inflammatory activity	[45]
eugenol	in vivo	inhibitor of $\alpha3\beta4$ nAChR	[82]
	in vitro	negative allosteric modulator of 5-HT_3 receptor	[97]
	in vitro	TRPV1 biphasic agonist	[137]
	in vitro	TRPA1 activator	[168]
	in vitro	TRPM8 agonist	[161]
	in vitro	K2P2.1 activator	[199]
	in vitro	Nav1.5 inhibitor	[212]
	ex vivo	TMEM16A inhibitor	[230,231]
flavonoids	in vitro	anti-inflammatory activity	[45]
	in vivo	anti-inflammatory activity	[45]

	human	anti-inflammatory activity	[45]
galanolactone	in vitro	antagonist of 5-HT_{3A} receptor	[101]
	in vivo	antagonist of 5-HT_{3A} receptor	[102–104]
	human	antagonist of 5-HT_{3A} receptor	[105–107]
gallic acid	in vitro	increase of gastric acid production	[65]
genistein	in vitro	increase of gastric acid production through PPARγ activation	[70]
gentiopicroside	human	w/o increase in plasma level of PYY	[37]
	ex vivo	L-type calcium channel inhibitor	[93]
geranial	in vitro	TRPV1 biphasic agonist	[137]
geraniol	in vitro	TRPM8 agonist	[185]
	in vitro	K2P2.1 activator	[199]
geranyl acetate	in vitro	TRPA1 activator	[172]
gingerol (unspecified)	in vivo	5-HT production decrease	[107]
	in vivo	5-HT receptor expression decrease	[107]
	in vivo	NK-1 receptor expression decrease	[301]
	in vivo	substance P production decrease	[301]
	in vivo	dopamine production decrease	[301]
	in vivo	D_2 receptor expression decrease	[301]
6-gingerol	in vitro	antagonist of 5-HT_{3A} receptor	[101]
	in vivo	antagonist of 5-HT_{3A} receptor	[102–104]
	human	antagonist of 5-HT_{3A} receptor	[105–107]
	in vitro	TRPV1 biphasic agonist	[133]
	in vitro	TRPA1 activator	[169]
	in vitro	TRPC5 inhibitor	[169]
	in vitro	Nav1.8 inhibitor	[206]
	ex vivo	M_3 receptor antagonist	[298]
8-gingerol	in vitro	antagonist of 5-HT_{3A} receptor	[101]
	in vivo	antagonist of 5-HT_{3A} receptor	[102–104]
	human	antagonist of 5-HT_{3A} receptor	[105–107]
	ex vivo	M_3 receptor antagonist	[298]
	in vivo	L-type calcium channel inhibitor	[304]
10-gingerol	in vitro	antagonist of 5-HT_{3A} receptor	[101]
	in vivo	antagonist of 5-HT_{3A} receptor	[102–104]
	human	antagonist of 5-HT_{3A} receptor	[105–107]
	in vivo	carboxylesterase inhibitor	[111]
	in vivo	α_2-adrenergic receptor antagonist	[219]
	in vivo	M_3 receptor antagonist	[298]
glabridin	in vitro	competitive antagonist of 5-HT_{3A} receptor	[100]
	in vivo	KCa1.1 activator	[201]
glycycoumarin	human	butyrylcholinesterase inhibitor	[111]
	in vitro	TRPM8 antagonist	[171]

	in vivo	α_2-adrenergic receptor antagonist	[219]
hesperidin	in vivo	antagonist of of 5-HT_{2C} receptor	[110]
	in vivo	agonist of 5-HT_4 receptor	[112]
	in vivo	mucus production increase	[203]
hispidulin	in vitro	TRPM8 antagonist	[171]
chlorogenic acid	ex vivo	competitive antagonist of AChR	[87]
chrysophanol	ex vivo	β_2-adrenergic receptor inhibitor	[279]
iridoids (aglycones)	in vitro	anti-inflammatory activity	[45]
	in vivo	anti-inflammatory activity	[45]
	human	anti-inflammatory activity	[45]
isocohumulone	in vitro	increase of gastric acid production through PPARγ activation	[67]
isohumulone	in vitro	increase of gastric acid production through PPARγ activation	[67]
isoliquiritigenin	in vivo	L-type calcium channel inhibitor	[92]
	in vivo	antagonist of 5-HT_{2C} receptor	[110]
	in vivo	TRPA1 activator	[171]
	in vitro	TRPC5 inhibitor	[195]
	in vitro	Nav1.7 inhibitor	[205]
	in vitro	Nav1.8 inhibitor	[206]
isopulegole	in vitro	TRPM8 agonist	[185]
kaempferol	in vitro	H^+/K^+-ATPase inhibitor	[22]
	in vitro	TRPC5 inhibitor	[194]
LCFA	in vitro	GPR40 receptor activation	[281]
licochalcone A	in vitro	antagonist of 5-HT_{3A} receptor	[100]
	in vitro	TRPM8 antagonist	[171]
linalool	in vivo	inhibitor of $\alpha3\beta4$ nAChR	[82]
	in vitro	negative allosteric modulator of 5-HT_3 receptor	[98]
	in vitro	TRPA1 activator	[177]
	in vitro	TRPM8 agonist	[185]
liquiritigenin	in vitro	non-competitive antagonist of 5-HT_{3A} receptor	[100]
	in vitro	TMEM16A inhibitor	[217]
liquiritin	in vitro	TRPV1 antagonist	[140]
loganic acid	human	w/o increase in plasma level of PYY	[37]
luteolin	in vitro	H^+/K^+-ATPase inhibitor	[22]
	in vitro	TMEM16A inhibitor	[233]
luteolin-7-glucoside	in vitro	H^+/K^+-ATPase inhibitor	[22]
marrubenol	ex vivo	L-type calcium channels inhibitor	[90]
menthol	in vitro	negative allosteric modulator of $\alpha4\beta2$ nAChR	[78]
	in vitro	competitive antagonist of $\alpha7$ nAChR	[79]
	ex vivo	L-type calcium channel inhibitor	[91]
	in vitro	negative allosteric modulator of 5-HT_3 receptor	[96]

	in vitro	TRPV1 antagonist	[141]
	in vitro	TRPA1 activator	[164]
	in vivo	TRPM8 agonist	[184]
	in vitro	K2P2.1 activator	[199]
	in vitro	Nav1.8 inhibitor	[207]
menthone	in vitro	TRPM8 agonist	[166]
myrcene	in vitro	TRPV1 biphasic agonist	[137]
	in vitro	TRPA1 activator	[165]
naringenin	in vitro	increase of gastric acid production through PPARγ activation	[69]
	in vitro	TRPV1 antagonist	[137]
oleanolic acid	in vitro	TRPV1 biphasic antagonist	[139]
oleic acid	in vivo	PGE_2	[262]
	in vivo	PPARα or PPARγ activation	[284–286]
6-paradol	in vitro	TRPV1 biphasic agonist	[134]
	in vitro	TRPA1 activator	[170]
pelargonidin	in vitro	H^+/K^+-ATPase inhibitor	[22]
polysaccharides (fermentable)	in vivo	SCFAs as FFAr2, and FFAr3 agonists	[256–261]
	in vitro	SCFAs as ClC-2 activators	[262]
polysaccharides (non-digestible)	in vivo	PIEZO2 activator	[253,254]
procyanidin B2	in vitro	CFTR channel inhibitor	[234]
protopine	ex vivo	competitive antagonist of AChR	[83]
	in vitro	Nav1.5, and Nav1.7 inhibitor	[211]
quercetagetin	in vitro	H^+/K^+-ATPase inhibitor	[22]
quercetin	in vitro	H^+/K^+-ATPase inhibitor	[22]
	ex vivo	competitive antagonist of AChR	[86]
	in vitro	TRPV1 antagonist	[137]
	in vitro	TRPM7 inhibitor	[191]
	in vitro	TRPC5 inhibitor	[194]
	in vitro	TMEM16A inhibitor	[233]
	in vitro	KCNQ1 modulator	[237]
quercetin-3-galactoside	in vitro	H^+/K^+-ATPase inhibitor	[22]
quercetin-3-gluco-rhamnoside	in vitro	H^+/K^+-ATPase inhibitor	[22]
quercetin-3-glucoside	in vitro	H^+/K^+-ATPase inhibitor	[22]
quercetin-3-rhamnoside	in vitro	H^+/K^+-ATPase inhibitor	[22]
quinine (hydrochloride)	human	no CCK excretion	[34–36]
	human	increase in plasma level of PYY	[35]
	human	various plasma level of GLP-1	[33,35,40,41]
resveratrol	in vitro	increase of gastric acid production through PPARγ activation	[69]
rhein	in vivo	CFTR channel activation	[276]

	in vivo	Na^+/K^+-ATPase inhibitor	[278]
	ex vivo	β_2-adrenergic receptor inhibitor	[279]
ricinoleic acid	in vitro	Na^+/K^+-ATPase inhibitor	[287]
	in vitro	active electrolytes absorption decrease	[288]
	in vivo	intestinal mucosa impairment	[289]
	ex vivo	eNOS activation	[277]
	ex vivo	chloride anion secretion	[291]
	in vivo	EP_3 agonist	[279]
	in vivo	? PGE_2	[279,280]
6-shogaol	in vitro	antagonist of 5-HT_{3A} receptor	[101]
	in vivo	antagonist of 5-HT_{3A} receptor	[102–104]
	human	antagonist of 5-HT_{3A} receptor	[105–107]
	in vitro	TRPV1 biphasic agonist	[134]
	in vitro	TRPA1 activator	[169]
	in vitro	TRPC5 inhibitor	[169]
	in vitro	Nav1.8	[206]
	in vivo	α_2-adrenergic receptor antagonist	[219]
	ex vivo	M_3 receptor antagonist	[298]
	ex vivo	? TRPA1 activator	[299]
8-shogaol	in vivo	α_2-adrenergic receptor antagonist	[219]
10-shogaol	ex vivo	TRPA1 activator	[134]
sweroside	human	w/o increase in plasma level of PYY	[37]
swertiamarin	human	w/o increase in plasma level of PYY	[37]
	ex vivo	L-type calcium channel inhibitor	[94]
tannic acid (*penta*-1,2,3,4,6-*O*-galloyl-*β*-D-glucose)	in vitro	H^+/K^+-ATPase inhibitor	[23]
	in vitro	TMEM16A inhibitor	[232]
	in vitro	CFTR channel inhibitor	[235]
	in vitro	KCNQ1/KCNE3 inhibitor	[236]
	in vitro	KCNQ2/3 activator	[236]
taxifolin	in vitro	H^+/K^+-ATPase inhibitor	[22]
thymol	in vitro	K2P2.1 activator	[199]
ursolic acid	ex vivo	competitive antagonist of AChR	[84]
	in vitro	TRPV1 (biphasic) antagonist	[137,139]
	in vivo	mucus production increase	[224]
verbascoside	in vitro	H^+/K^+-ATPase inhibitor	[20]
	in vitro	anti-inflammatory activity	[45]
	in vivo	anti-inflammatory activity	[45]
	human	anti-inflammatory activity	[45]
	ex vivo	competitive antagonist of AChR	[69]
vitexin	in vitro	TRPV1 antagonist	[137]

zingerone	in vitro	antagonist of 5-HT_{3A} receptor	[101]
	in vivo	antagonist of 5-HT_{3A} receptor	[102–104]
	human	antagonist of 5-HT_{3A} receptor	[105–107]
	in vitro	TRPV1 biphasic agonist	[133]
	in vitro	TRPA1 activator	[169]
	in vitro	TRPC5 inhibitor	[169]

Abbreviations: A2 receptor – adenosine 2 receptor; AQPs – aquaporins; α_2-adrenergic receptor – alfa 2-adrenergic receptor; β_2-adrenergic receptor – beta 2-adrenergic receptor; BK (= KCa1.1 = KCNMA1) channel – big potassium channel, calcium-activated potassium channel subfamily M alpha 1; CCK – cholecystokinin; CFTR – cystic fibrosis transmembrane conductance regulator protein; ClC-2 – chloride channel 2; D_2 receptor – dopamine receptor D_2; eNOS – endothelial nitric oxide synthase; EP_3 receptor – prostaglandin EP_3 receptor; FFAR2, FFAR3 – free fatty acid receptors 2 and 3; GLP-1 – glucagon-like peptide-1; GPR40 receptor – G protein-coupled receptor 40; 5-HT_{2C}, 5-HT_3, 5-HT_{3A}, 5-HT_4 receptors – 5-hydroxytryptamine (serotonin) receptors 5-HT_{2C}, 5-HT_3, 5-HT_{3A}, 5-HT_4 subtypes; K2P2.1 (KCNK2, TREK-1), – potassium channels (potassium channel subfamilies K member 2); KCNQ (KCNQ1, KCNQ2, KCNQ3) – potassium voltage-gated channels subfamily Q member 1, 2, 3; KCNQ1/KCNE3 complex – complex of potassium voltage-gated channel subfamily Q member 1 and potassium voltage-gated channel subfamily E regulatory subunit 3; LCFA – long chain fatty acid; M_3 receptor (mAChR) – muscarinic acetylcholine receptor M_3; nAChRs (α3β4 nAChRs, α4β2 nAChRs, α7 nAChRs) – nicotinic acetylcholine receptors type α3β4, α4β2, α7; Nav1.2, Nav1.3, Nav1.5, Nav1.6, Nav1.7, Nav1.8 channels – voltage-gated sodium channels 1.2, 1.3, 1.5, 1.6, 1.7, 1.8; NK-1 – neurokinin-1 receptor; PGE_2 – prostaglandin E_2; PIEZO2-type mechanoreceptor – piezo type mechanosensitive ion channel component 2; PPAR (PPARα, PPARγ) – peroxisome proliferator-activated receptors α, γ; H^+/K^+-ATPase inhibitors (PPIs) – proton pump inhibitors; PYY – peptide YY; SCFA – short-chain fatty acid; TAS2R – bitter taste receptor; TMEM16A (ANO1) – transmembrane 16 (Anoctamin 1) protein mediates calcium-activated chloride channel (CaCC); TRP channels (superfamilies TRPA1, TRPC5, TRPM8, TRPV1) – transient receptor potential (TRP) channels.
